# Synthesis and Evaluation of Isosteviol Derivatives: Promising Anticancer Therapies for Colon Cancer

**DOI:** 10.3390/biomedicines13040793

**Published:** 2025-03-25

**Authors:** Yecang Chen, Feifei Zhu, Yuxin Ding, Lin Xing, Enxiao Wang, Yixiang Fang, Ruilong Sheng, Qidong Tu, Ruihua Guo

**Affiliations:** 1 College of Food Science and Technology, Shanghai Ocean University, Shanghai 201306, China; zff476004403@163.com (F.Z.); dingyuxin0305@163.com (Y.D.); xinglin0220@163.com (L.X.); enxiaowang7374@163.com (E.W.); yxfang2025@163.com (Y.F.); 2 Division of Biomedical Engineering, James Watt School of Engineering, University of Glasgow, Glasgow G12 8LT, UK; 2495282c@student.gla.ac.uk; 3 CQM—Centro de Química da Madeira, Campus da Penteada, Universidade da Madeira, 9000-390 Funchal, Portugal; ruilong.sheng@staff.uma.pt; 4 Jiangxi Provincial Key Laboratory of Drug Design and Evaluation, School of Pharmacy, Jiangxi Science & Technology Normal University, Nanchang 330013, China; 5 Marine Biomedical Science and Technology Innovation Platform of Lin-Gang Special Area, Shanghai 201306, China; 6 Department of Marine Biopharmacology, College of Food Science and Technology, Shanghai Ocean University, Shanghai 201306, China

**Keywords:** isosteviol, anticancer, colon cancer, structure-activity relationships, molecular docking, TP53

## Abstract

**Background:** Isosteviol, a tetracyclic diterpenoid with a beyerene-type skeleton, exhibited wide pharmacological activities and an inhibitory impact on tumor proliferation in colon cancer; **Methods:** 22 isosteviol derivatives were synthesized by modifying the C-16 and C-19 position of isosteviol, and then the inhibitory activities of derivatives **2**–**22** were evaluated by CCK8 method. Next, the structure–activity relationships (SARs) of these isosteviol derivatives in HCT116 cells were discussed in detail. Network pharmacology was employed to predict and analyze the targets of isosteviol in the treatment of colon cancer; **Results:** The results indicated derivative **8** possessed stronger inhibitory activity against HCT116 and HepG2 cells (IC_50_ = 6.20 ± 0.61 μM for HCT116, and IC_50_ = 39.84 ± 0.43 μM for HepG2). Additionally, cell cycle analysis indicated that derivative **8** arrested HCT116 cells at the G1 phase and increased the percentage of apoptotic cells. Moreover, the molecular docking showed that derivative **8** could interact with TP53 through its Tyr-1600 and Leu-1534 residues (docking energy: −11.84 kcal/mol); **Conclusions:** With these results, we can conclude that derivative **8** may be a promising candidate for anticancer chemotherapy.

## 1. Introduction

Colon cancer is one of the most common malignancies worldwide and is regarded as a critical public health issue, remaining a leading cause of cancer-related mortality [1,2]. According to the 2023 data, it ranks among the top three in the United States in terms of morbidity and mortality rates [3]. Based on population-based data, the 5-year survival rate for metastatic colorectal cancer is 14% [4]. Clinically, surgical resection remains the main treatment approach for localized tumors. For advanced tumors, chemotherapy and radiotherapy are employed to shrink the tumor and control symptoms, despite their side effects [5]. Drugs such as fluorouracil, leucovorin calcium, oxaliplatin, and irinotecan (FOLFOXIRI), along with bevacizumab (triplet + bev), are the standard multi-agent chemotherapy regimens for the first-line treatment of colon cancer. However, these regimens are applicable only to patients with good performance status [6]. However, patients who received triplet + bev were more likely to have a grade ≥3 and serious treatment-emergent adverse events, suggesting an increased risk of toxicity with triplet chemotherapy [7,8,9]. Although current drugs have certain therapeutic effects on colon cancer, colon cancer is still a growing threat to human health worldwide, especially in developing countries where patient survival and prognosis have been improved but are still unsatisfactory [10]. Therefore, researchers have focused on identifying new anticancer agents with low cytotoxicity for the treatment of colorectal cancer [4,11,12].

**Figure 1:**
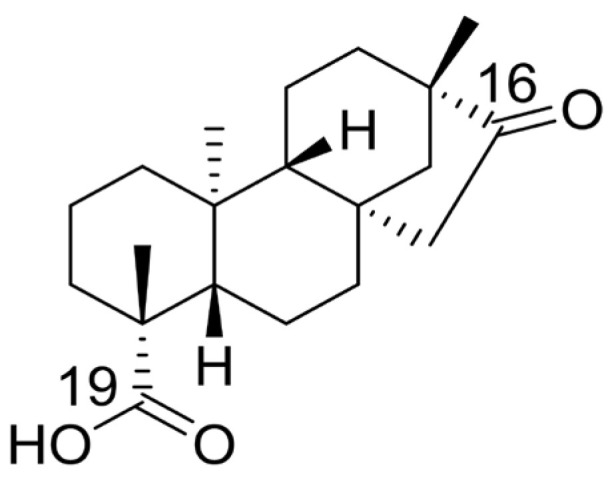
The chemical structure of isosteviol.

Isosteviol (Figure 1), a tetracyclic diterpenoid with a beyerene-type skeleton, demonstrates wide pharmacological activities including anti-tumor [13], lowering blood glucose [14], anti-hypertension [15], anti-inflammatory [16], and other pharmacological activities [17,18]. The ketone group in the D ring, along with the carboxyl group at the C-19 position, serve as ideal sites for structural modification in isosteviol [18]. Heterocyclic compounds are extensively used in therapeutics owing to their unique physicochemical and pharmacological properties [19,20]. A large number of heterocyclic compounds available on the pharmaceutical market exhibit significant anticancer activity, including 5-fluorouracil, methotrexate, doxorubicin, and zorubicin [21,22]. Therefore, in this study, we introduced different piperazine fragments at the C-19 position of isosteviol and structurally modified its D-ring by introducing different benzene rings and furan rings as well as thiophene at the C-16 position. Our aim was to discover new compounds with high activity and selectivity for small-molecule anticolonic activity. Based on a literature review on isosteviol, two cancer cell lines (HCT116 and HepG2) were screened to evaluate the biological activity of isosteviol derivatives. Among these, isosteviol derivatives demonstrated significant activity on HCT116 cells, prompting further in-depth investigation.

## 2. Materials and Methods

### 2.1. General Methods

All the reagents and materials were purchased from commercial suppliers and used without further purification. Isosteviol was obtained from acid-hydrolysis of stevioside, which was extracted from the plant *Stevia rebaudiana.* Thin-layer chromatography (TLC) and silica gel column chromatography (300–400 mesh) were achieved using instruments from Qingdao Makall Group Co., Ltd. (Qingdao, China). ^1^H NMR and ^13^C NMR spectra were recorded on a Bruker DRX-500^1^H/^13^C, 500 MHz/125 MHz) spectrometer (Bruker, Bremerhaven, Germany) and chemical shifts were given in δ (ppm) with tetramethyl silane (TMS) as an internal reference. High-resolution mass spectra (HRMS) were measured on an Advantage Max LCQ Thermo-Finnigan mass spectrometer.

### 2.2. Synthesis

#### 2.2.1. General Procedure for Preparation of Derivative **1**

To a solution of isosteviol (200 mg, 0.628 mmol) in N,N-dimethylformamide (DMF) (1.5 mL), bromopropane (87 μL, 0.942 mmol) and K_2_CO_3_ (130.2 mg, 0.942 mmol) were added at room temperature. After stirring for 9 h, the reaction mixture was diluted with water and extracted with ethyl acetate (3 × 30 mL). The organic layer was dried over anhydrous Na_2_SO_4_, filtered, and the filtrate was evaporated under reduced pressure. The crude material was subjected to column chromatography using petroleum ether/ethyl acetate (15:1) as the eluent, affording pure derivative **1** as a white amorphous powder. Yield: 70.2%.

#### 2.2.2. General Procedure for Preparation of Derivative **2**

To a solution of derivative **1** (200 mg, 0.56 mmol) in C_2_H_5_OH (2 mL) was added NH_2_OH·HCl (60 mg, 0.84 mmol) and K_2_CO_3_ (116.1 mg, 0.84 mmol). After refluxing for 8 h, the reaction mixture was diluted with water and extracted with ethyl acetate (3 × 30 mL). The organic layer was dried over anhydrous Na_2_SO_4_, filtered, and the filtrate was evaporated under reduced pressure. The crude material was subjected to column chromatography using petroleum ether/ethyl acetate (6:1) as an eluent and afforded pure derivative **2** as a white amorphous powder. Yield: 70.8%.

#### 2.2.3. General Procedure for Preparation of Derivative **3**

To a solution of derivative **2** (50 mg, 0.13 mmol) in DMF (1.5 mL) with EDCI (124.6 mg, 0.65 mmol) and DMAP (3.18 mg, 0.026 mmol) was added benzoic acid (31.8 mg, 0.26 mmol). After refluxing for 6 h, the reaction mixture was diluted with water and extracted with ethyl acetate (3 × 30 mL). The organic layer was collected and dried over anhydrous Na_2_SO_4_, filtered, and the filtrate was evaporated under reduced pressure. Finally, the residue was purified by column chromatography over silica gel to obtain the pure target derivative **3**.

*propyl(4R,4aS,6aR,9S,11aR,11bS,E)-8-((benzoyloxy)imino)-4,9,11b-trimethyltetradecahydro-6a,9-methanocyclohepta[a]naphthalene-4-carboxylate* (**3**). White powder, yield: 45.6% (eluent: petroleum ether/ethyl acetate, 6:1, *v*/*v*). ^1^H NMR (500 MHz, CDCl_3_) δ: 8.15 (d, J = 8.1 Hz, 2H), 7.74 (d, J = 8.3 Hz, 2H), 4.05–3.94 (m, 2H), 2.23–2.14 (m, 2H), 1.95–1.89 (m, 1H), 1.84–1.74 (m, 4H), 1.73–1.63 (m, 6H), 1.62–1.48 (m, 4H), 1.34–1.30 (m, 2H), 1.28–1.27 (m, 3H), 1.20 (s, 3H), 1.17–1.13 (m, 1H), 1.12–1.08 (m, 1H), 1.05–1.01 (m, 1H), 0.97 (t, J = 7.4 Hz, 3H), 0.94–0.89 (m, 1H), 0.76 (s, 3H) (Figure S1). ^13^C NMR (125 MHz, CDCl_3_) δ: 179.92, 177.50, 162.95, 132.96, 130.08, 125.70, 125.68, 125.66, 66.06, 57.23, 56.31, 54.65, 45.34, 44.00, 41.02, 40.75, 39.98, 39.49, 38.83, 38.22, 37.94, 29.25, 22.14, 22.00, 21.71, 20.46, 19.17, 13.65, 10.88 (Figure S2). HRMS (ESI, *m*/*z*) calcd. for C_30_H_41_NO_4_ [M + H]^+^: 480.3116; found: 479.1000.

#### 2.2.4. General Procedure for Preparation of Derivatives **4**–**13**

To a solution of derivative **2** (50 mg, 0.13 mmol) in N,N-dimethylformamide (DMF) (1.5 mL) with EDCI (124.6 mg, 0.65 mmol) and DMAP (3.18 mg, 0.026 mmol) was added 2-furancarboxylic acid (29.1 mg, 0.26 mmol), 5-bromo-2-furancarboxylic acid (49.7 mg, 0.26 mmol), 5-chloro-2-furancarboxylic acid (38.1 mg, 0.26 mmol), 5-methylfuran-2-carboxylic acid (32.8 mg, 0.26 mmol), 5-nitro-2-furancarboxylic acid (40.8 mg, 0.26 mmol), 2-thiophenecarboxylic acid (33.3 mg, 0.26 mmol), 5-bromo-2-thiophenecarboxylic acid (53.8 mg, 0.26 mmol), 5-chloro-2-thiophenecarboxylic acid (42.3 mg, 0.26 mmol), 5-methylthiophene-2-carboxylic acid (37.0 mg, 0.26 mmol), and 5-nitrothiophene-2-carboxylic acid (45.0 mg, 0.26 mmol), respectively, at room temperature. After stirring for 6 h, the reaction mixture was diluted with water and extracted with ethyl acetate (3 × 30 mL). The organic layer was collected and dried over anhydrous Na_2_SO_4_, filtered, and the filtrate was evaporated under reduced pressure. Finally, the residue was purified by column chromatography over silica gel to obtain the pure target derivatives **4**–**13**.

*Propyl(4R,4aS,6aR,9S,11aR,11bS,E)-8-(((furan-2-carbonyl)oxy)imino)-4,9,11b-trimethyltetradecahydro-6a,9-methanocyclohepta[a]naphthalene-4-carboxylate* (**4**). White powder, yield: 50.2% (eluent: petroleum ether/ethyl acetate, 8:1, *v*/*v*). ^1^H NMR (500 MHz, CDCl_3_) δ: 7.64–7.61 (m, 1H), 7.21 (dd, J = 3.5, 0.6 Hz, 1H), 6.54 (dd, J = 3.5, 1.7 Hz, 1H), 4.05–3.95 (m, 2H), 2.21–2.16 (m, 2H), 1.93–1.88 (m, 1H), 1.83–1.73 (m, 3H), 1.71–1.62 (m, 6H), 1.57–1.41 (m, 4H), 1.33–1.27 (m, 3H), 1.25 (s, 3H), 1.20 (s, 3H), 1.15–1.07 (m, 2H), 0.98 (dd, J = 9.0, 5.9 Hz, 3H), 0.92–0.85 (m, 1H), 0.76 (s, 3H) (Figure S3). ^13^C NMR (125 MHz, CDCl_3_) δ: 179.51, 177.55, 156.62, 146.69, 143.64, 118.34, 111.95, 66.03, 57.26, 56.39, 54.71, 45.24, 44.01, 40.93, 40.77, 40.00, 39.45, 38.83, 38.20, 37.97, 29.25, 22.14, 22.02, 21.72, 20.46, 19.17, 13.62, 10.88 (Figure S4). HRMS (ESI, *m*/*z*) calcd. for C_28_H_39_NO_5_ [M + H]^+^: 470.2908; found: 470.1991.

*Propyl(4R,4aS,6aR,9S,11aR,11bS,E)-8-(((5-bromofuran-2-carbonyl)oxy)imino)-4,9,11b-trimethyltetradecahydro-6a,9-methanocyclohepta[a]naphthalene-4-carboxylate* (**5**). White powder, yield: 60.4% (eluent: petroleum ether/ethyl acetate, 10:1, *v*/*v*). ^1^H NMR (500 MHz, CDCl_3_) δ: 7.09 (d, J = 3.3 Hz, 1H), 6.12 (d, J = 3.2 Hz, 1H), 4.02–3.95 (m, 2H), 2.38 (s, 3H), 2.19–2.09 (m, 2H), 1.96–1.86 (m, 2H), 1.81–1.75 (m, 2H), 1.75–1.71 (m, 1H), 1.67–1.61 (m, 4H), 1.61–1.56 (m, 2H), 1.54–1.50 (m, 1H), 1.47–1.41 (m, 3H), 1.28–1.24 (m, 2H), 1.22 (s, 3H), 1.17 (s, 3H), 1.02–0.98 (m, 1H), 0.95 (t, J = 7.6 Hz, 4H), 0.90–0.84 (m, 1H), 0.73 (s, 3H) (Figure S5). ^13^C NMR (125 MHz, CDCl_3_) δ: 179.10, 177.52, 157.74, 156.70, 141.78, 119.79, 108.50, 65.96, 57.16, 56.32, 54.63, 45.11, 43.92, 40.83, 40.70, 39.92, 39.35, 38.70, 38.12, 37.90, 29.16, 22.09, 21.94, 21.66, 20.38, 19.09, 14.14, 13.53, 10.83 (Figure S6). HRMS (ESI, *m*/*z*) calcd. for C_28_H_38_BrNO_5_ [M + H]^+^: 548.2013; found: 548.1859.

*Propyl(4R,4aS,6aR,9S,11aR,11bS,E)-8-(((5-chlorofuran-2-carbonyl)oxy)imino)-4,9,11b-trimethyltetradecahydro-6a,9-methanocyclohepta[a]naphthalene-4-carboxylate* (**6**). White powder, yield: 67.3% (eluent: petroleum ether/ethyl acetate, 8:1, *v*/*v*). ^1^H NMR (500 MHz, CDCl_3_) δ: 7.18 (dd, J = 3.5, 1.2 Hz, 1H), 6.33 (d, J = 3.5 Hz, 1H), 4.03–3.93 (m, 2H), 2.20–2.10 (m, 2H), 1.91–1.86 (m, 1H), 1.80–1.74 (m, 3H), 1.72–1.57 (m, 6H), 1.54–1.39 (m, 4H), 1.31–1.25 (m, 2H), 1.22 (s, 3H), 1.17 (s, 3H), 1.13–1.06 (m, 2H), 1.03–0.99 (m, 1H), 0.96 (t, J = 6.8 Hz, 3H), 0.91–0.84 (m, 1H), 0.73 (s, 3H) (Figure S7). ^13^C NMR (125 MHz, CDCl_3_) δ: 179.72, 177.52, 155.53, 142.73, 141.53, 120.47, 109.05, 65.99, 57.10, 56.25, 54.54, 45.24, 43.91, 40.86, 40.64, 39.90, 39.34, 38.73, 38.11, 37.89, 29.13, 22.06, 21.93, 21.65, 20.36, 19.06, 13.52, 10.86 (Figure S8). HRMS (ESI, *m*/*z*) calcd. for C_28_H_38_ClNO_5_ [M + H]^+^: 504.2519; found: 504.0534.

*Propyl(4R,4aS,6aR,9S,11aR,11bS,E)-4,9,11b-trimethyl-8-(((5-methylfuran-2-carbonyl)oxy)imino)tetradecahydro-6a,9-methanocyclohepta[a]naphthalene-4-carboxylate* (**7**). White powder, yield: 65.3% (eluent: petroleum ether/ethyl acetate, 8:1, *v*/*v*). ^1^H NMR (500 MHz, CDCl_3_) δ: 7.13 (d, J = 3.5 Hz, 1H), 6.46 (d, J = 3.5 Hz, 1H), 4.01–3.91 (m, 2H), 2.17–2.07 (m, 2H), 1.89–1.83 (m, 1H), 1.78–1.68 (m, 4H), 1.67–1.61 (m, 4H), 1.60–1.54 (m, 1H), 1.51–1.48 (m, 1H), 1.46–1.36 (m, 3H), 1.28–1.24 (m, 2H), 1.20 (s, 3H), 1.15 (s, 3H), 1.11–1.03 (m, 2H), 1.00–0.96 (m, 1H), 0.93 (t, J = 7.4 Hz, 3H), 0.89–0.81 (m, 1H), 0.71 (s, 3H) (Figure S9). ^13^C NMR (125 MHz, CDCl_3_) δ: 179.62, 177.40, 155.36, 145.06, 127.83, 120.48, 113.99, 65.89, 57.00, 56.16, 54.45, 45.15, 43.81, 40.78, 40.55, 39.81, 39.26, 38.64, 38.02, 37.81, 29.05, 22.00, 21.86, 21.57, 20.28, 18.98, 13.44, 10.82 (Figure S10). HRMS (ESI, *m*/*z*) calcd. for C_29_H_41_NO_5_ [M + H]^+^: 484.3065; found: 484.0853.

*Propyl(4R,4aS,6aR,9S,11aR,11bS,E)-4,9,11b-trimethyl-8-(((5-nitrofuran-2-carbonyl)oxy)imino)tetradecahydro-6a,9-methanocyclohepta[a]naphthalene-4-carboxylate* (**8**). White powder, yield: 65.3% (eluent: petroleum ether/ethyl acetate, 8:1, *v*/*v*). ^1^H NMR (500 MHz, CDCl_3_) δ: 7.28 (d, J = 3.8 Hz, 1H), 7.13 (d, J = 3.8 Hz, 1H), 3.93 (d, J = 7.9 Hz, 2H), 2.17 (s, 3H), 1.88 (d, J = 4.6 Hz, 2H), 1.85 (s, 3H), 1.77 (s, 4H), 1.75 (s, 2H), 1.71 (s, 2H), 1.34 (s, 3H), 1.24 (s, 3H), 1.18 (s, 6H), 0.95 (s, 3H), 0.75 (s, 3H) (Figure S11). ^13^C NMR (125 MHz, CDCl_3_) δ: 177.25, 175.53, 131.27, 130.41, 119.94, 117.17, 112.09, 66.03, 57.22, 56.87, 56.69, 45.85, 40.23, 40.06, 40.02, 39.95, 39.19, 37.98, 37.45, 35.30, 28.88, 23.39, 21.99, 20.10, 19.98, 19.01, 17.75, 10.82 (Figure S12). HRMS (ESI, *m*/*z*) calcd. for C_28_H_38_N_2_O_7_ [M+H]^+^: 515.2759; found: 515.2929.

*Propyl(4R,4aS,6aR,9S,11aR,11bS,E)-4,9,11b-trimethyl-8-(((thiophene-2-carbonyl)oxy)imino)tetradecahydro-6a,9-methanocyclohepta[a]naphthalene-4-carboxylate* (**9**). White powder, yield: 61.6% (eluent: petroleum ether/ethyl acetate, 5:1, *v*/*v*). ^1^H NMR (500 MHz, CDCl_3_) δ: 7.83 (dd, J = 3.7, 1.2 Hz, 1H), 7.57 (dd, J = 5.0, 1.2 Hz, 1H), 7.11 (dd, J = 4.9, 3.8 Hz, 1H), 4.02–3.93 (m, 2H), 2.19–2.10 (m, 2H), 1.91–1.85 (m, 1H), 1.81–1.73 (m, 3H), 1.72–1.57 (m, 6H), 1.54–1.50 (m, 1H), 1.48–1.43 (m, 2H), 1.42–1.37 (m, 1H), 1.30–1.25 (m, 2H), 1.23 (s, 3H), 1.17 (s, 3H), 1.13–1.05 (m, 2H), 1.02–0.98 (m, 1H), 0.96 (t, J = 7.4 Hz, 3H), 0.90–0.83 (m, 1H), 0.73 (s, 3H) (Figure S13). ^13^C NMR (125 MHz, CDCl_3_) δ: 179.13, 177.44, 159.79, 133.79, 132.60, 132.15, 127.82, 65.93, 57.05, 56.19, 54.52, 45.02, 43.85, 40.80, 40.61, 39.85, 39.35, 38.66, 38.07, 37.85, 29.09, 22.03, 21.89, 21.60, 20.32, 19.02, 13.42, 10.88 (Figure S14). HRMS (ESI, *m*/*z*) calcd. for C_28_H_39_NO_4_S [M+H]^+^: 486.2680; found: 486.3421.

*Propyl(4R,4aS,6aR,9S,11aR,11bS,E)-8-(((5-bromothiophene-2-carbonyl)oxy)imino)-4,9,11b-trimethyltetradecahydro-6a,9-methanocyclohepta[a]naphthalene-4-carboxylate* (**10**). White powder, yield: 54.9% (eluent: petroleum ether/ethyl acetate, 5:1, *v*/*v*). ^1^H NMR (500 MHz, CDCl_3_) δ: 7.67 (d, J = 3.7 Hz, 1H), 6.79 (d, J = 3.7 Hz, 1H), 4.02–3.98 (m, 2H), 2.54 (s, 3H), 2.21–2.10 (m, 2H), 1.93–1.86 (m, 1H), 1.82–1.73 (m, 4H), 1.70–1.65 (m, 3H), 1.63–1.58 (m, 1H), 1.55–1.40 (m, 5H), 1.31–1.27 (m, 2H), 1.24 (s, 4H), 1.19 (s, 3H), 1.14–1.07 (m, 2H), 0.98 (t, J = 5.1 Hz, 3H), 0.92–0.85 (m, 1H), 0.75 (s, 3H) (Figure S15). ^13^C NMR (125 MHz, CDCl_3_) δ: 178.92, 177.57, 159.90, 148.30, 134.39, 129.43, 126.51, 66.04, 57.19, 56.31, 54.66, 45.05, 43.96, 40.87, 40.74, 39.96, 39.42, 38.72, 38.17, 37.96, 29.19, 22.12, 21.98, 21.69, 20.42, 19.12, 15.87, 13.54, 10.97 (Figure S16). HRMS (ESI, *m*/*z*) calcd. for C_28_H_38_BrNO_4_S [M+H]^+^: 564.1785; found: 564.1370.

*Propyl(4R,4aS,6aR,9S,11aR,11bS,E)-8-(((5-chlorothiophene-2-carbonyl)oxy)imino)-4,9,11b-trimethyltetradecahydro-6a,9-methanocyclohepta[a]naphthalene-4-carboxylate* (**11**). White powder, yield: 65.2% (eluent: petroleum ether/ethyl acetate, 5:1, *v*/*v*). ^1^H NMR (500 MHz, CDCl_3_) δ: 7.66 (d, J = 4.0 Hz, 1H), 6.96 (d, J = 4.0 Hz, 1H), 4.00 (td, J = 6.5, 1.0 Hz, 2H), 2.22–2.08 (m, 2H), 1.93–1.86 (m, 1H), 1.82–1.65 (m, 8H), 1.63–1.58 (m, 1H), 1.55–1.52 (m, 1H), 1.50–1.45 (m, 2H), 1.44–1.40 (m, 1H), 1.31–1.27 (m, 2H), 1.24 (s, 3H), 1.19 (s, 3H), 1.14–1.07 (m, 2H), 0.99 (dd, J = 9.0, 5.8 Hz, 3H), 0.92–0.83 (m, 2H), 0.75 (s, 3H) (Figure S17). ^13^C NMR (125 MHz, CDCl_3_) δ: 179.42, 177.54, 159.00, 137.81, 133.68, 130.17, 127.36, 66.08, 57.14, 56.24, 54.57, 45.20, 43.95, 40.92, 40.69, 39.95, 39.43, 38.73, 38.17, 37.96, 29.15, 22.09, 21.99, 21.68, 20.40, 19.09, 13.53, 10.98 (Figure S18). HRMS (ESI, *m*/*z*) calcd. for C_28_H_38_ClNO_4_S [M+H]^+^: 520.2290; found: 520.0744.

*Propyl(4R,4aS,6aR,9S,11aR,11bS,E)-4,9,11b-trimethyl-8-(((5-methylthiophene-2-carbonyl)oxy)imino)tetradecahydro-6a,9-methanocyclohepta[a]naphthalene-4-carboxylate* (**12**). White powder, yield: 60.2% (eluent: petroleum ether/ethyl acetate, 5:1, *v*/*v*). ^1^H NMR (500 MHz, CDCl_3_) δ: 7.62 (d, J = 4.0 Hz, 1H), 7.10 (d, J = 4.0 Hz, 1H), 4.01 (t, J = 6.4 Hz, 2H), 2.22–2.09 (m, 2H), 1.94–1.87 (m, 1H), 1.83–1.66 (m, 8H), 1.64–1.58 (m, 1H), 1.56–1.40 (m, 4H), 1.32–1.27 (m, 2H), 1.24 (s, 3H), 1.19 (s, 3H), 1.15–1.08 (m, 2H), 1.00 (dd, J = 9.3, 5.5 Hz, 4H), 0.92–0.85 (m, 1H), 0.75 (s, 3H) (Figure S19). ^13^C NMR (125 MHz, CDCl_3)_ δ: 179.44, 177.57, 158.91, 134.37, 133.13, 131.02, 120.77, 66.10, 57.15, 56.26, 54.59, 45.22, 43.96, 40.94, 40.71, 39.97, 39.44, 38.75, 38.18, 37.98, 29.17, 22.11, 22.01, 21.70, 20.42, 19.11, 13.55, 11.02 (Figure S20). HRMS (ESI, *m*/*z*) calcd. for C_29_H_41_NO_4_S [M+H]^+^: 500.2836; found: 500.2084.

*Propyl(4R,4aS,6aR,9S,11aR,11bS,E)-4,9,11b-trimethyl-8-(((5-nitrothiophene-2-carbonyl)oxy)imino)tetradecahydro-6a,9-methanocyclohepta[a]naphthalene-4-carboxylate* (**13**). White powder, yield: 57.6% (eluent: petroleum ether/ethyl acetate, 5:1, *v*/*v*). ^1^H NMR (500 MHz, CDCl_3_) δ: 7.89 (d, J = 4.3 Hz, 1H), 7.76 (d, J = 4.3 Hz, 1H), 4.02–3.97 (m, 2H), 2.21–2.12 (m, 2H), 1.93–1.88 (m, 1H), 1.82–1.76 (m, 3H), 1.74–1.64 (m, 5H), 1.64–1.47 (m, 5H), 1.46–1.40 (m, 1H), 1.34–1.30 (m, 1H), 1.24 (s, 3H), 1.18 (s, 3H), 1.16–1.07 (m, 3H), 0.98 (t, J = 7.4 Hz, 3H), 0.92–0.86 (m, 1H), 0.75 (s, 3H) (Figure S21). ^13^C NMR (125 MHz, CDCl_3_) δ: 180.31, 177.40, 158.36, 155.62, 136.91, 132.27, 127.96, 66.02, 56.98, 56.08, 54.38, 45.39, 43.84, 40.93, 40.52, 39.84, 39.33, 38.71, 38.07, 37.84, 29.04, 21.96, 21.90, 21.58, 20.30, 18.98, 13.44, 10.85 (Figure S22). HRMS (ESI, *m*/*z*) calcd. for C_28_H_38_N_2_O_6_S [M+H]^+^: 531.2531; found: 531.0218.

#### 2.2.5. General Procedure for Preparation of Derivative **14**

To a solution of isosteviol (200 mg, 0.628 mmol) in N,N-dimethylformamide (DMF) (1.5 mL) was added 1,2-dibromoethane (81 μL, 0.942 mmol) and K_2_CO_3_ (130.2 mg, 0.942 mmol) at room temperature. After stirring for 9 h, the reaction mixture was diluted with water and extracted with ethyl acetate (3 × 30 mL). The organic layer was dried over anhydrous Na_2_SO_4_, filtered, and the filtrate was evaporated under reduced pressure. The crude material was subjected to column chromatography using petroleum ether/ethyl acetate (5:1) as an eluent and afforded pure derivative **14** as a white amorphous powder. Yield: 56.4%.

#### 2.2.6. General Procedure for Preparation of Derivatives **15**–**18**

To a solution of derivative **14** (50 mg, 0.118 mmol) in N,N-dimethylformamide (DMF) (1.5 mL) with K_2_CO_3_ (24.46 mg, 0.177 mmol) was added piperazine (81 mg, 0.94 mmol), N-methylpiperazine (23.64 mg, 0.236 mmol), N-ethylpiperazine (26.95 mg, 0.236 mmol), N-propylpiperazine (30.26 mg, 0.236 mmol), and N-butylpiperazine (33.57 mg, 0.236mmol), respectively, at room temperature. After stirring for 12 h, the reaction mixture was diluted with water and extracted with ethyl acetate (3 × 30 mL). The organic layer was collected and dried over anhydrous Na_2_SO_4_, filtered, and the filtrate was evaporated under reduced pressure. Finally, the residue was purified by column chromatography over silica gel to obtain the pure target derivatives **15**–**18**.

*2-(piperazin-1-yl) ethyl (4R,4aS,6aR,9S,11aR,11bS)-4,9,11b-trimethyl-8-oxotetra decahydro-6a,9-methanocyclohepta[a]naph thalene-4-carboxylate* (**15**). Brown powder, yield: 57.6% (eluent: DCM/MeOH, 5:1, *v*/*v*). ^1^H NMR (500 MHz, CDCl_3_) δ: 4.17–4.11 (m, 2H), 3.46 (s, 2H), 2.95 (s, 1H), 2.72–2.58 (m, 5H), 2.50 (s, 4H), 2.18–2.13 (m, 1H), 1.89–1.83 (m, 1H), 1.82–1.72 (m, 3H), 1.71–1.62 (m, 3H), 1.61–1.57 (m, 1H), 1.55–1.51 (m, 1H), 1.49–1.43 (m, 1H), 1.42–1.37 (m, 2H), 1.37–1.33 (m, 1H), 1.24 (s, 1H), 1.17 (s, 4H), 1.13–1.09 (m, J = 11.8 Hz, 1H), 1.03–0.98 (m, 1H), 0.96 (s, 3H), 0.93–0.85 (m, 1H), 0.70 (s, 3H) (Figure S23). ^13^C NMR (125 MHz, CDCl_3_) δ: 222.64, 177.34, 61.65, 57.22, 56.60, 54.82, 54.39, 53.32, 51.55, 48.82, 48.75, 43.89, 41.66, 39.95, 39.58, 38.16, 38.08, 37.42, 29.07, 21.81, 20.44, 19.97, 19.03, 13.54 (Figure S24). HRMS (ESI, *m*/*z*) calcd. for C_26_H_42_N_2_O_3_ [M+H]^+^: 431.3224; found: 431.3237.

*2-(4-methylpiperazin-1-yl)ethyl(4R,4aS,6aR,9S,11aR,11bS)-4,9,11b-trimethyl-8-oxotetradecahydro-6a,9-methanocyclohepta[a]naphthalene-4-carboxylate* (**16**). White powder, yield: 63.8% (eluent: DCM/MeOH, 15:1, *v*/*v*). ^1^H NMR (500 MHz, CDCl_3_) δ: 4.19–4.07 (m, 2H), 3.45 (s, 2H), 2.64–2.45 (m, 8H), 2.44–2.36 (m, 1H), 2.32 (s, 3H), 2.14 (d, J = 13.3 Hz, 1H), 1.89–1.75 (m, 3H), 1.74–1.55 (m, 5H), 1.55–1.44 (m, 2H), 1.44–1.18 (m, 5H), 1.17 (s, 3H), 1.15–1.07 (m, 1H), 1.03–0.97 (m, 1H), 0.96 (s, 3H), 0.88 (td, J = 13.1, 3.9 Hz, 1H), 0.69 (s, 3H) (Figure S25). ^13^C NMR (125 MHz, CDCl_3)_ δ: 222.74, 177.34, 61.45, 57.15, 56.44, 55.05 (2C), 54.75, 54.33, 52.91 (2C), 48.81, 48.72, 45.85, 43.88, 41.61, 39.90, 39.56, 38.13, 38.04, 37.39, 29.05, 21.79, 20.41, 19.94, 19.00, 13.53 (Figure S26). HRMS (ESI, *m*/*z*) calcd. for C_27_H_44_N_2_O_3_ [M+H]^+^: 445.3432; found: 445.8410.

*2-(4-ethylpiperazin-1-yl)ethyl(4R,4aS,6aR,9S,11aR,11bS)-4,9,11b-trimethyl-8-oxotetradecahydro-6a,9-methanocyclohepta[a]naphthalene-4-carboxylate* (**17**). White powder, yield: 67.9% (eluent: DCM/MeOH, 12:1, *v*/*v*). ^1^H NMR (500 MHz, CDCl_3_) δ: 4.20–4.06 (m, 2H), 2.66–2.53 (m, 7H), 2.48–2.41 (m, 3H), 2.29 (s, 2H), 2.19–2.10 (m, 1H), 1.89–1.83 (m, 1H), 1.82–1.72 (m, 2H), 1.72–1.56 (m, 5H), 1.55–1.44 (m, 2H), 1.44–1.32 (m, 3H), 1.27–1.19 (m, 2H), 1.17 (s, 3H), 1.15–1.11 (m, 1H), 1.09 (t, J = 7.2 Hz, 3H), 1.03–0.98 (m, 1H), 0.96 (s, 3H), 0.92–0.84 (m, 1H), 0.70 (s, 3H) (Figure S27). ^13^C NMR (125 MHz, CDCl_3_) δ: 222.76, 177.34, 61.55, 57.17, 56.51, 54.76, 54.34, 53.05 (2C), 52.79 (2C), 52.40, 48.82, 48.72, 43.88, 41.62, 39.91, 39.56, 38.14, 38.05, 37.40, 29.05, 21.80, 20.41, 19.95, 19.00, 13.53, 11.83 (Figure S28). HRMS (ESI, *m*/*z*) calcd. for C_28_H_46_N_2_O_3_ [M+H]^+^: 459.3588; found: 458.6365.

*2-(4-propylpiperazin-1-yl)ethyl(4R,4aS,6aR,9S,11aR,11bS)-4,9,11b-trimethyl-8-oxotetradecahydro-6a,9-methanocyclohepta[a]naphthalene-4-carboxylate (**18**)*. White powder, yield: 70.1% (eluent: petroleum ether/ethyl acetate, 4:1, *v*/*v*). ^1^H NMR (500 MHz, CDCl_3_) δ: 4.19–4.08 (m, 2H), 2.65–2.43 (m, 10H), 2.33–2.28 (m, 2H), 2.18–2.12 (m, 1H), 1.88–1.63 (m, 7H), 1.63–1.56 (m, 2H), 1.55–1.44 (m, 4H), 1.41–1.37 (m, 2H), 1.37–1.32 (m, 1H), 1.23 (s, 2H), 1.17 (s, 3H), 1.12–1.08 (m, 1H), 1.03–0.98 (m, 1H), 0.96 (s, 3H), 0.88 (t, J = 7.4 Hz, 4H), 0.70 (s, 3H) (Figure S29). ^13^C NMR (125 MHz, CDCl_3_) δ: 222.69, 177.32, 61.58, 60.71, 57.18, 56.56, 54.78, 54.36, 53.25 (2C), 53.20 (2C), 48.82, 48.71, 43.87, 41.64, 39.92, 39.56, 38.14, 38.05, 37.41, 29.06, 21.80, 20.42, 19.97, 19.01, 13.53, 12.05 (Figure S30). HRMS (ESI, *m*/*z*) calcd. for C_29_H_48_N_2_O_3_ [M+H]^+^: 473.3745; found: 472.7200.

#### 2.2.7. General Procedure for Preparation of Derivatives **19**–**22**

To a solution of derivative **15** (100 mg, 0.232 mmol) in N,N-dimethylformamide (DMF) (1.5 mL) with K_2_CO_3_ (48.1 mg, 0.348 mmol) was added 2-chlorobenzylbromide (59.6 μL, 0.464 mmol), 2-bromobenzylbromide (61.0 μL, 0.464 mmol), 2-iodobenzyl bromide (65.6 μL, 0.464 mmol), and 1-bromo-4-nitrobenzene (55.1 μL, 0.464 mmol), respectively, at room temperature. After stirring for 6 h, the reaction mixture was diluted with water and extracted with ethyl acetate (3 × 30 mL). The organic layer was collected and dried over anhydrous Na2SO4, filtered, and the filtrate was evaporated under reduced pressure. Finally, the residue was purified by column chromatography over silica gel to obtain the pure target derivatives **19**–**22.**

*2-(4-(2-chlorobenzyl)piperazin-1-yl)ethyl(4R,4aS,6aR,9S,11aR,11bS)-4,9,11b-trimethyl-8-oxotetradecahydro-6a,9-methanocyclohepta[a]naphthalene-4-carboxylate* (**19**). White powder, yield: 57.8% (eluent: petroleum ether/ethyl acetate, 6:1, *v*/*v*). ^1^H NMR (500 MHz, CDCl_3_) δ: 7.77–7.68 (m, 1H), 7.34–7.27 (m, 1H), 7.24–7.21 (m, 1H), 6.89–6.80 (m, 1H), 4.13–3.99 (m, 2H), 3.43 (s, 2H), 2.58–2.54 (m, 2H), 2.53–2.39 (m, 8H), 2.11–2.04 (m, 1H), 1.83–1.57 (m, 7H), 1.57–1.49 (m, 2H), 1.49–1.36 (m, 4H), 1.31–1.25 (m, 1H), 1.20–1.15 (m, 2H), 1.11–1.10 (m, 3H), 1.06–1.04 (m, 1H), 0.96–0.92 (m, 1H), 0.89 (s, 3H), 0.85–0.79 (m, 1H), 0.63 (s, 3H) (Figure S31). ^13^C NMR (125 MHz, CDCl_3_) δ: 222.54, 177.16, 140.42, 139.39, 130.30, 128.61, 127.90, 100.65, 66.32, 61.48, 56.98, 56.38, 54.58, 54.16, 53.28 (2C), 52.88 (2C), 48.63, 48.53, 43.69, 41.44, 39.72, 39.39, 37.96, 37.87, 37.22, 28.90, 21.62, 20.24, 19.81, 18.84, 13.38 (Figure S32). HRMS (ESI, *m*/*z*) calcd. for C_33_H_47_ClN_2_O_3_ [M+H]^+^: 555.3355; found: 555.3415.

*2-(4-(2-bromobenzyl)piperazin-1-yl)ethyl(4R,4aS,6aR,9S,11aR,11bS)-4,9,11b-trimethyl-8-oxotetradecahydro-6a,9-methanocyclohepta[a]naphthalene-4-carboxylate* (**20**). White powder, yield: 60.3% (eluent: petroleum ether/ethyl acetate, 5:1, *v*/*v*). ^1^H NMR (500 MHz, CDCl_3_) δ: 7.54–7.49 (m, 1H), 7.46–7.42 (m, 1H), 7.29–7.23 (m, 1H), 7.08 (t, J = 7.6 Hz, 1H), 4.20–4.07 (m, 2H), 3.59 (s, 2H), 2.65–2.61 (m, 2H), 2.60–2.42 (m, 8H), 2.18–2.13 (m, 1H), 1.90–1.64 (m, 7H), 1.64–1.56 (m, 2H), 1.56–1.44 (m, 2H), 1.42–1.37 (m, 2H), 1.37–1.31 (m, 1H), 1.26–1.18 (m, 2H), 1.17 (s, 3H), 1.13–1.08 (m, 1H), 1.03–0.98 (m, 1H), 0.96 (s, 3H), 0.92–0.85 (m, 1H), 0.70 (s, 3H) (Figure S33). ^13^C NMR (125 MHz, CDCl_3_) δ: 222.62, 177.27, 137.53, 132.80, 130.87, 128.46, 127.24, 124.78, 61.79, 61.60, 57.14, 56.51, 54.74, 54.32, 53.40 (2C), 53.13 (2C), 48.77, 48.64, 43.83, 41.59, 39.87, 39.52, 38.10, 38.01, 37.36, 29.04, 21.76, 20.38, 19.94, 18.98, 13.50 (Figure S34). HRMS (ESI, *m*/*z*) calcd. for C_33_H_47_BrN_2_O_3_ [M+H]^+^: 599.2850; found: 588.3996.

*2-(4-(2-iodobenzyl)piperazin-1-yl)ethyl(4R,4aS,6aR,9S,11aR,11bS)-4,9,11b-trimethyl-8-oxotetradecahydro-6a,9-methanocyclohepta[a]naphthalene-4-carboxylate* (**21**). White powder, yield: 54.9% (eluent: petroleum ether/ethyl acetate, 5:1, *v*/*v*). ^1^H NMR (500 MHz, CDCl_3_) δ: 7.43 (dd, J = 7.5, 1.4 Hz, 1H), 7.34–7.29 (m, 1H), 7.21 (td, J = 7.4, 1.2 Hz, 1H), 7.15 (td, J = 7.6, 1.7 Hz, 1H), 4.19–4.07 (m, 2H), 3.60 (s, 2H), 2.65–2.44 (m, 10H), 2.17–2.12 (m, 1H), 1.89–1.63 (m, 7H), 1.62–1.55 (m, 2H), 1.55–1.43 (m, 2H), 1.41–1.36 (m, 2H), 1.36–1.30 (m, 1H), 1.26–1.18 (m, 2H), 1.16 (s, 3H), 1.12–1.07 (m, 1H), 1.03–0.98 (m, 1H), 0.96 (s, 3H), 0.91–0.84 (m, 1H), 0.69 (s, 3H) (Figure S35). ^13^C NMR (125 MHz, CDCl_3_) δ: 222.60, 177.27, 135.79, 134.42, 130.88, 129.50, 128.20, 126.63, 61.60, 59.26, 57.14, 56.51, 54.74, 54.32, 53.38 (2C), 53.14 (2C), 48.76, 48.65, 43.83, 41.59, 39.87, 39.52, 38.10, 38.01, 37.37, 29.04, 21.77, 20.38, 19.95, 18.98, 13.50 (Figure S36). HRMS (ESI, *m*/*z*) calcd. for C_33_H_47_IN_2_O_3_ [M+H]^+^: 647.2711; found: 647.1413.

*2-(4-(4-nitrophenyl)piperazin-1-yl)ethyl(4R,4aS,6aR,9S,11aR,11bS)-4,9,11b-trimethyl-8-oxotetradecahydro-6a,9-methanocyclohepta[a]naphthalene-4-carboxylate* (**22**). Brown powder, yield: 50.3% (eluent: petroleum ether/ethyl acetate, 4:1, *v*/*v*). ^1^H NMR (500 MHz, CDCl_3_) δ: 8.09 (d, J = 6.3 Hz, 2H), 6.81 (d, J = 9.4 Hz, 2H), 4.25–4.12 (m, 2H), 3.41–3.37 (m, 4H), 2.66 (t, J = 5.8 Hz, 2H), 2.65–2.61 (m, 4H), 2.59 (d, J = 3.7 Hz, 1H), 2.19–2.13 (m, 1H), 1.91–1.85 (m, 1H), 1.83–1.75 (m, 2H), 1.72–1.65 (m, 3H), 1.63–1.61 (m, 1H), 1.61–1.56 (m, 1H), 1.55–1.51 (m, 1H), 1.48 (dd, J = 13.6, 3.8 Hz, 1H), 1.44–1.38 (m, 3H), 1.23 (s, 1H), 1.19 (s, 4H), 1.15–1.10 (m, 1H), 1.05–0.98 (m, 1H), 0.95 (s, 3H), 0.94–0.86 (m, 1H), 0.71 (s, 3H) (Figure S37). ^13^C NMR (125 MHz, CDCl_3_) δ: 222.63, 177.30, 154.91, 138.55, 126.05 (2C), 112.80 (2C), 61.09, 57.12, 56.52, 54.70, 54.30, 52.81 (2C), 48.80, 48.63, 47.17 (2C), 43.92, 41.58, 39.86, 39.55, 38.14, 38.02, 37.37, 29.13, 21.81, 20.41, 19.94, 19.02, 13.62.32, 19.85, 18.93, 13.54 (Figure S38). HRMS (ESI, *m*/*z*) calcd. for C_32_H_45_N_3_O_5_ [M+H]^+:^ 552.3439; found: 551.6603.

### 2.3. Biological Evaluation and Pharmacological Mechanisms

#### 2.3.1. Sample Pretreatment

All the synthesized isosteviol derivatives (and isosteviol control) were dissolved in DMSO to obtain 100 mM of original isosteviol derivative solution and stored in a refrigerator at −20 °C for reserve. Then, the original solution was diluted to 1 μM, 5 μM, 10 μM, 20 μM, and 30 μM by McCoy’s 5A or DMEM.

#### 2.3.2. Cell Culture and Treatments

McCoy’s 5A medium (McCoy’s 5A), high glucose Dulbecco’s modified Eagle medium (DMEM) and phosphate buffered saline (PBS) were purchased from Wisent (Nanjing, China). Fetal bovine serum (FBS), trypsin, penicillin, and streptomycin were purchased from Shanghai QiDa Biotechnology Co., Ltd. (Shanghai, China). The Cell Counting Kit-8 (CCK-8), cell cycle and apoptosis analysis kits were from MultiSciences Biotech Co., Ltd. (Hangzhou, China). Doxorubicin hydrochloride (DOX·HCl) with a purity of 98%, used as the positive control, was obtained from Shanghai Macklin Biochemical Technology Co., Ltd. (Shanghai, China). The cancer cell lines HCT116 and HepG2 were obtained from the American Type Culture Collection (ATCC, VA, USA). These cell lines were mycoplasma-free and have been authenticated using STR profiling. Cells were cultured in McCoy’s 5A (Wisent, Nanjing, China) or high glucose DMEM (Wisent, Nanjing, China) media supplemented with 10% FBS, penicillin, and streptomycin (100 U/mL) at 37 °C in a humidified incubator containing 5% CO_2_.

#### 2.3.3. Antiproliferative Activity In Vitro [23]

The antiproliferative activities of all isosteviol derivatives were evaluated against colorectal carcinoma cells (HCT116) and liver cancer cells (HepG2) by CCK-8 assay. For this assay, 100 μL (5 × 10^3^/mL) of cells per well were seeded in 96-well plates and allowed to incubate for 24 h. Then, the compounds with different concentrations were added. After 72 h of incubation, the CCK-8 solution was added, and the plates were incubated again for another 1 h at 37 °C. The absorbance at 450 nm was measured using a microplate reader (Spectramax Plus 384, Molecular Devices, Sunnyvale, CA, USA). The IC_50_ was determined by the Graph Pad Prism 8.0 software. Three independent experiments were conducted.

#### 2.3.4. Cell Cycle Assay [24]

The HCT116 cells in the logarithmic growth phase were digested into 6-well plates (1 × 10^6^ cells/well). After incubation for 24 h, cells were treated with different concentrations of derivative **8**, and the cells were incubated for 24 h. Then, the cells were collected and washed with PBS, and 1 mL DNA staining solution and 10 μL of Permeabilization solution (PI) (Hangzhou, China) were added, followed by vortex oscillation for 5–10 s and incubation at room temperature under the dark for 30 min. The DNA content analysis was carried out using a BD Accuri C6 flow cytometer (Becton & Dickinson Co., Miami, FL, USA), and the data were analyzed using the FlowJo 10.8.1 software.

#### 2.3.5. Cell Apoptosis Assay [24]

Apoptotic cells were measured by the Annexin V-APC/PI apoptosis detection kit (MultiSciences Biotech) according to the protocol described. Briefly, the HCT116 cells were treated with various concentrations of derivative **8** for 48 h of incubation at 37 °C. Then, the cells were harvested and resuspended in PBS at a concentration of 1 × 10^6^ cells/mL. After centrifugation at 1000 rpm for 5 min, 500 μL 1 × Binding Buffer, 5 μL APC-conjugated annexin V and 10 μL of annexin V-APC, and 10 μL of Permeabilization solution (PI) were added. The mixture was incubated for 5 min at room temperature in the absence of light. Following gentle vortexing, the sample was analyzed using a BD Accuri C6 flow cytometer (Becton & Dickinson Co.). The percentages of the apoptotic and necrotic cells for each sample were estimated. The percentages of apoptotic cells were analyzed using the FlowJo 10.8.1 software.

#### 2.3.6. Molecular Docking Studies [25]

The prepared crystal structure of TP53 (PDB code: 6VIP) was taken from the website https://www.rcsb.org/ for compound docking. Firstly, the preparation of the proteins was carried out, which mainly included adding hydrogen atoms, removing water molecules and optimizing the protein structure. Docking was performed using the AutoDock 4.2.6 software, and the results were analyzed using the Discovery Studio Visualizer 2024 software.

## 3. Results and Discussion

### 3.1. Chemistry

**Scheme 1:**
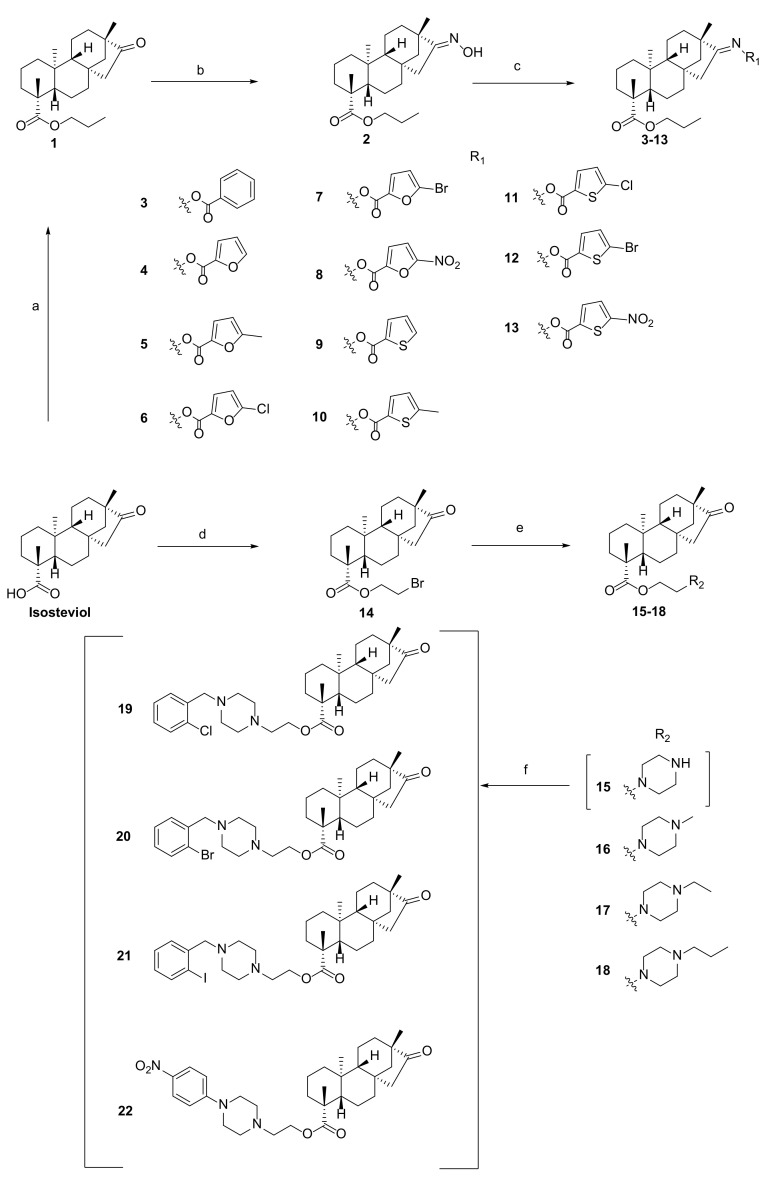
Reagents and conditions: (a) C_3_H_7_Br, K_2_CO_3_, DMF, r.t., 9 h, 70.2%; (b) NH_2_OH∙HCl, K_2_CO_3_, EtOH, 60 °C, 8 h, 70.8%; (c) EDCI, DMAP, acid, DMF, 60 °C, r.t., 6 h, 45.6–67.3%; (d) 1,2-dibromoethane, K_2_CO_3_, DMF, r.t., 9 h, 56.4%; (e) alkyl piperazine, K_2_CO_3_, DMF, r.t., 12 h, 57.6–70.1%; (f) R-X, K_2_CO_3_, DMF, 60 °C, 6 h, 50.3–60.3%.

A total of 22 isosteviol derivatives were synthesized following the synthesis route outlined in Scheme 1. Subsequently, their chemical structures were comprehensively characterized using ^1^H NMR, ^13^C NMR, and HRMS.

As shown in Scheme 1, the derivatives **3**–**13** were synthesized through a two-step process [26]. First, a propyl group was introduced at the C-19 position of isosteviol under base catalysis (K_2_CO_3_) conditions to obtain derivative **1**. Second, derivative **2** was synthesized by introducing an oxime at the C-16 position of derivative **1** in the presence of K_2_CO_3_ in ethanol at 60 °C. Subsequently, derivative **2** was then esterified under standard esterification conditions by heating in DMF with furanocarboxylic, thiophenecarboxylic, and benzoic acids bearing different substituents. The resulting ester derivatives **3**–**13** were obtained as white powder.

The nucleophilic reaction between isosteviol and 1,2-dibromoethane was carried out under alkaline conditions, generating an intermediate, **14**. Subsequently, intermediate **14** participated in a substitution reaction with a variety of alkyl piperazine rings in a basic milieu, ultimately yielding the target derivatives **15**–**18**. Under alkaline conditions, derivative **15** underwent nucleophilic reactions with bromobenzyl groups containing different substituents and 1-bromo-4-nitrobenzene in DMF, yielding the target derivatives **19**–**22**.

### 3.2. Biological Evaluation

#### 3.2.1. HCT116 Inhibitory Activity and Structure–Activity Relationship

**Figure 2:**
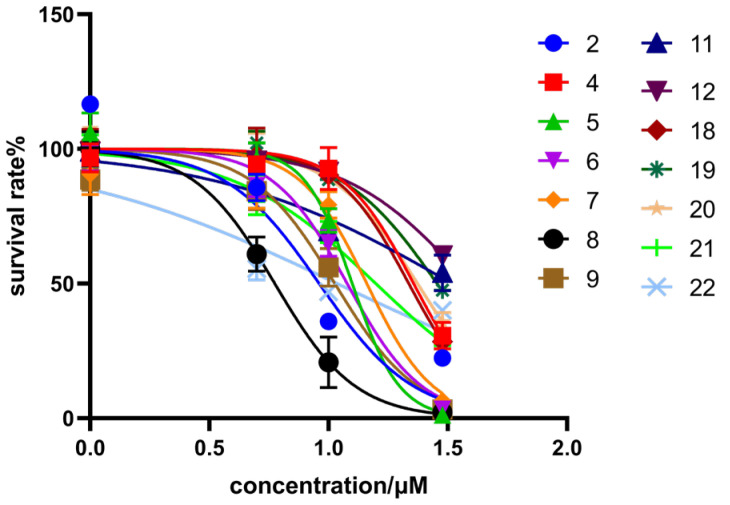
Cell viability of HCT116 cells treated with isosteviol derivatives.

All the synthesized isosteviol derivatives were evaluated for their antiproliferative effects against two human cancer cell lines (HCT116 and HepG2) using the CCK-8 assay, with DOX as the positive drug (Figure 2). The inhibitory activity of each isosteviol derivative was expressed as the concentration of the compound that achieved 50% inhibition (IC_50_) of the anticancer standard. Their IC_50_ values are presented in Table 1.

Isosteviol showed moderate inhibitory activity against HCT116 (IC_50_ = 24.80 ± 2.43 μM), but had no inhibitory activity against HepG2. Twenty-two isosteviol derivatives were synthesized based on chemical modifications on the functional groups at the C-16 and C-19 positions. The results showed that isosteviol derivatives possessed moderate to strong inhibitory activity against HCT116. Derivatives **3**, **10**, **13**, and **15**–**17** (IC_50_ > 30 μM) had no inhibitory activity. Derivatives **4** and **18**–**20** possessed moderate inhibitory activity against HCT116. Moreover, derivatives **2**, **5**–**9**, **11**, **12**, **21,** and **22** presented significant inhibitory potency against HCT116, with average IC_50_ values ranging from 6.20 to 15.77 μM. Among them, derivative **8** showed a remarkable inhibitory effect on HCT116 (IC_50_ = 6.20 ± 0.61 μM) and HepG2 (IC_50_ = 39.84 ± 0.43 μM).

Isosteviol derivatives exhibited the highest sensitivity to HCT116. Therefore, HCT116 was selected for the preliminary study of structure–activity relationships (SARs). The introduction of a propyl group to the C-19 position of isosteviol afforded ester derivative **1**. The oxime derivative **2** was yielded by treating isosteviol and derivative **1** with NH_2_OH·HCl and its inhibitory potency on HCT116 cells was evaluated. Derivative **2** showed stronger inhibitory activity against HCT116 (IC_50_ = 24.80 ± 2.43 μM vs. IC_50_ = 7.31 ± 1.12 μM) compared to isosteviol, indicating the introduction of an oxime group at the C-19 position of isosteviol enhanced inhibitory activity against HCT116.

In the next step, oxime ester derivative **3** was synthesized by introducing a benzene ring, and its inhibitory activity was evaluated. Nevertheless, derivative **3** had no inhibitory activity against HCT116. Furan and thiophene were introduced to the C-16 position of isosteviol to synthesize derivatives **4** and **9**, aiming to study the effect of different rings on inhibitory activity. Compared with derivatives **3** (IC_50_ > 30 μM) and **4** (IC_50_ = 22.75 ± 0.86 μM), derivative **9** (IC_50_ = 10.62 ± 1.21 μM) exhibited significantly stronger inhibitory activity against HCT116. The results showed that the introduction of thiophene to the C-16 position of isosteviol was helpful in improving the inhibitory activity against HCT116.

Introducing different substituents (CH_3_, Cl, Br, and NO_2_) to the C-4 position of the furan ring afforded the oxime ether derivatives **5**–**8**. Then, their inhibitory activities were evaluated. It can be observed that derivatives **5**–**8** had stronger inhibitory potency compared to derivative **4**, meaning that substituents at the C-4 position of the furan ring enhanced inhibitory activity. Comparison of the derivatives **5**–**8** revealed that the derivative with the electron-donating group (CH_3_) was less active than those with the electron-withdrawing groups (Cl, Br, and NO_2_). Meanwhile, the 4-NO_2_ derivative **8** (IC_50_ = 6.20 ± 0.61 μM) exhibited stronger inhibitory activity than derivatives **6** (IC_50_ = 14.47 ± 0.71 μM) and **7** (IC_50_ = 11.97 ± 0.91 μM), indicating that the introduction of NO_2_ to the C-4 position of the furan ring resulted in significantly stronger inhibitory activity against HCT116.

Derivatives **10**–**13** were synthesized by introducing different substituents (CH_3_, Cl, Br, and NO_2_) to the C-4 position of the thiophene ring, and their inhibitory activities were studied. The CH_3_ derivative **10** and the NO_2_ derivative **13** had no inhibitory activity (IC_50_ > 30 μM). Derivatives **9** (IC_50_ = 10.62 ± 1.21 μM), **11** (IC_50_ = 10.44 ± 0.84 μM) and **12** (IC_50_ = 10.00 ± 0.65 μM) exhibited similar inhibitory activity. However, among them, the Br derivative **12** had relatively stronger inhibitory activity. The results indicated that the introduction of Br to the C-4 position of the thiophene ring showed significantly stronger inhibitory activity against HCT116.

Derivative **14** was synthesized by introducing bromopropane at the C-19 position of isosteviol. Then, by introducing piperazine in different extension chains to derivative **14**, derivatives **15**–**18** were obtained. Derivatives **15**–**17** had no inhibitory activity. However, when the branched chain was extended to three carbon atoms, the inhibitory activity increased. The results indicated that the inhibitory activity increased as the side chain length reached three carbon atoms.

To investigate whether the introduction of a benzyl group was favorable in increasing the inhibitory activity of derivative **17**, different substituents (Cl, Br, I, and NO_2_) were introduced on benzyl to obtain derivatives **19**–**22**. Then, their inhibitory activities were studied. The 2-I derivative **21** (IC_50_ = 15.77 ± 0.69 μM) showed stronger inhibitory activity than the 2-Cl derivative **19** (IC_50_ = 28.50 ± 0.14 μM) and 2-Br derivative **20** (IC_50_ = 23.61 ± 0.63 μM). However, the 2-Cl derivative **19** showed lower inhibitory activity than isosteviol. The 4-NO_2_ derivative **22** (IC_50_ = 10.85 ± 0.26 μM) exhibited significantly stronger inhibitory activity than derivatives **19**–**21**. It can be observed that the introduction of a 4-NO_2_ on the benzyl of derivative **17** was favorable for enhancing inhibitory activity against HCT116.

**Figure 3:**
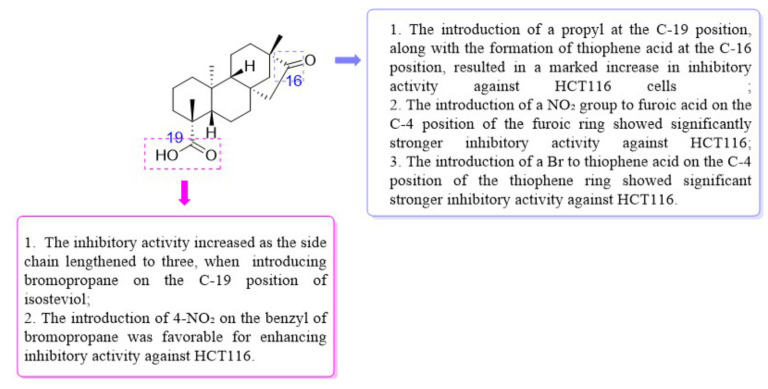
The SARs of isosteviol derivatives for inhibitory activity on HCT116 cells.

From the SARs (Figure 3), the inhibitory activity of HCT116 was influenced by modifications at the C-16 and C-19 positions. Delightfully, derivative **8** possessed stronger inhibitory activity against HCT116 and HepG2 cells (IC_50_ = 6.20 ± 0.61 μM for HCT116, and IC_50_ = 39.84 ± 0.43 μM for HepG2). Therefore, isosteviol derivative **8** was chosen as a model compound for further studies on the biological mechanism of the HCT116 cell line.

#### 3.2.2. Effects of Derivative **8** on Cell Cycle Distribution

**Figure 4:**
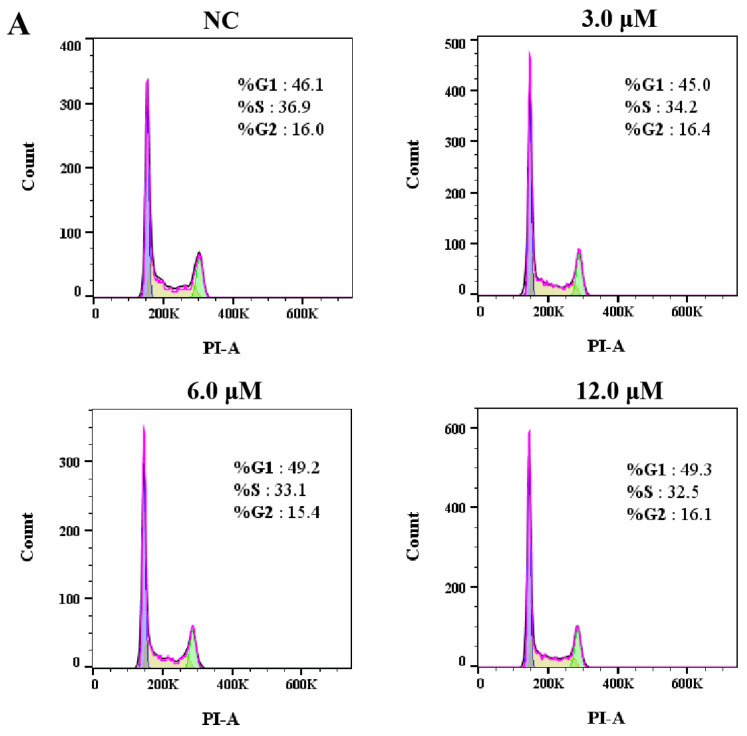

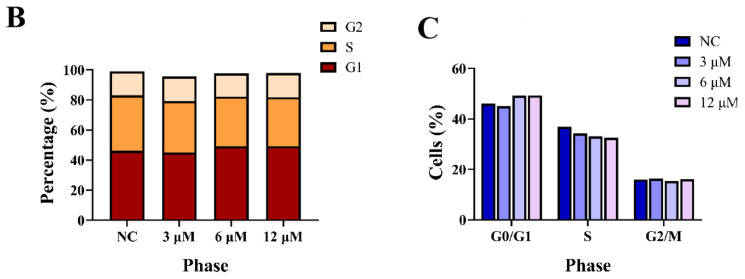
(**A**) Cell cycle analysis of HCT116 cells treated with derivative **8**. (**B**) Effects of derivative **8** on HCT116 cell cycle phase distribution at different concentrations. (**C**) Trends of HCT116 cell cycle phases in the presence of different concentrations of derivative **8**.

We next investigated the effect of derivative **8** on the cell cycle in HCT116 cells. The G1 phase serves as a preparatory stage for DNA replication, the S phase facilitates DNA synthesis, and the G2 phase ensures that the cell is ready for division. Collectively, these phases play a critical role in genetic stability and the orderly progression of the cell cycle. Accordingly, tumor cells were subjected to treatment with derivative **8** at three different concentrations (3.0, 6.0, and 12.0 μM) for 24 h, and DMSO-treated cells served as the negative control (NC). The treated cells were incubated with propidium iodide (PI). Subsequently, the fraction of tumor cells in the G1 phase exhibited a dose-dependent increase upon treatment with derivative **8** (Figure 4). Additionally, compared to the control group, derivative **8** caused a substantial decrease in the number of cells in the S phase. The proportion of cells in the G1 phase increased from 46.1% (NC) to 45.0% (3.0 μM), 49.2% (6.0 μM), and 49.3% (12.0 μM). Meanwhile, the proportion of cells in the S phase decreased from 36.9% (NC) to 34.2% (3.0 μM), 33.1% (6.0 μM), and 32.5% (12.0 μM). The results indicated that derivative **8** had an influence on the cell cycle at the G1 phase arrest. As changes in cell viability are the result of alterations in both cell proliferation and survival, we next evaluated the impact of derivative **8** on tumor cell apoptosis.

#### 3.2.3. The Effect of Derivative **8** on Apoptosis

**Figure 5:**
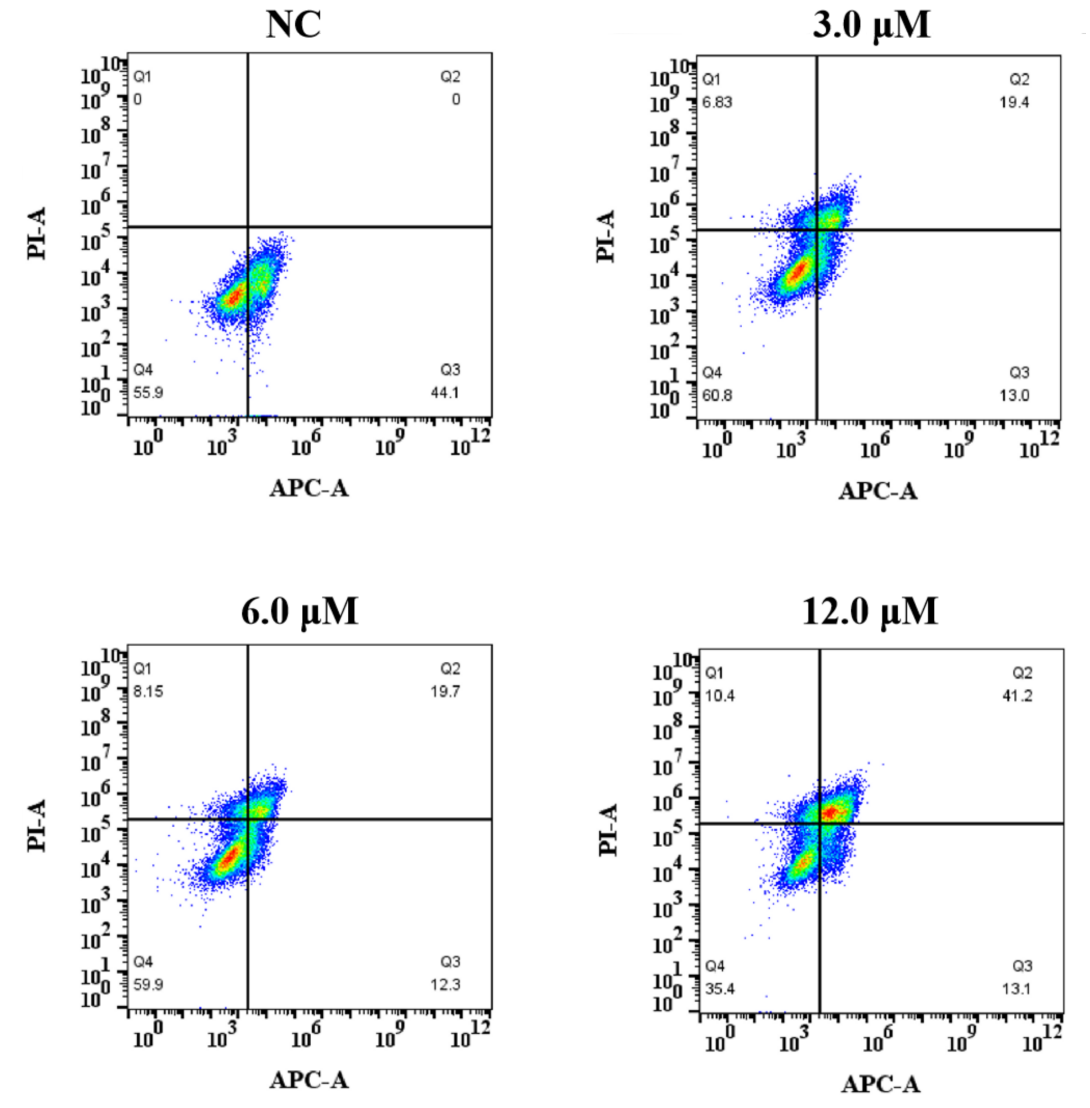
Effect of derivative **8** on the percentage of annexin V-APC-positive staining on HCT116 cells at distinct concentrations (3.0, 6.0, and 12.0 μM) for 48 h.

In this study, the annexin V-APC/PI flow cytometry assay was used to verify whether derivative **8**-mediated inhibition of growth and proliferation was associated with apoptosis. HCT116 cell lines were treated with derivative **8** at the concentrations of 3.0, 6.0, and 12.0 μM for 48 h (Figure 5 and Table 2). Derivative **8** significantly induced apoptosis against HCT116 cells in a dose-dependent manner. Using untreated cells as the NC, the apoptotic effect of derivative **8** was observed on the HCT116 cell line. The early-stage apoptosis rates of derivative **8** gradually decreased from 44.1% (NC) to 13.0%, 12.3%, and 13.1%. After treatment with derivative **8** at the concentrations of 3.0, 6.0, and 12.0 μM, respectively, the late-stage apoptosis rates changed from 19.4% and 19.7% to 41.2%. The proportions of apoptotic HCT116 cells treated with derivative **8** were 32.4% (3.0 μM), 32.0% (6.0 μM), and 54.3% (12.0 μM). The results indicated that derivative **8** induced late apoptosis, resulting in a remarkable increase in apoptotic cells in a concentration-dependent manner.

### 3.3. Network Pharmacology

#### 3.3.1. Characterization of Potential Protein Targets of the Natural Product Isosteviol

A total of 16,253 potential protein targets for isosteviol were retrieved from the BATMAN-TCM database (bionet.ncpsb.org.cn/batman-tcm/index.php, accessed on 12 February 2025). These potential targets for isosteviol included ESRRG, COX4I1, MPC1, etc.

#### 3.3.2. Compound–Target–Disease Network

**Figure 6:**
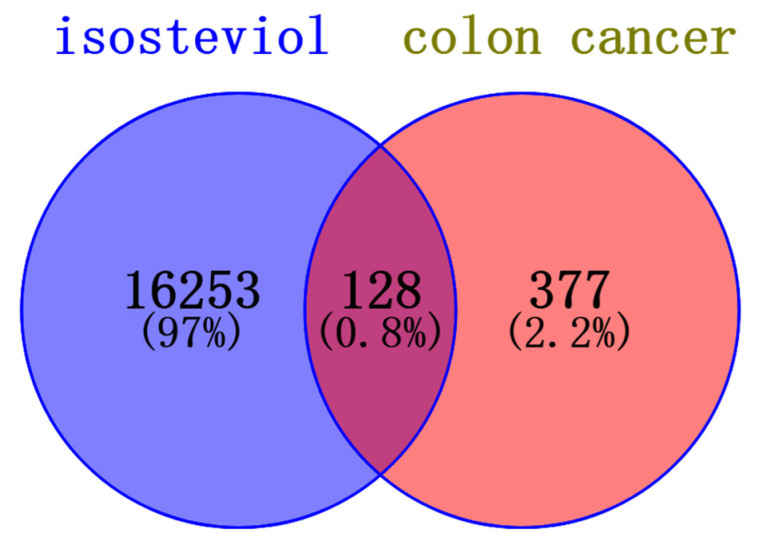
Venn diagram representing the intersection of protein targets common to both isosteviol and colon cancer.

**Figure 7:**
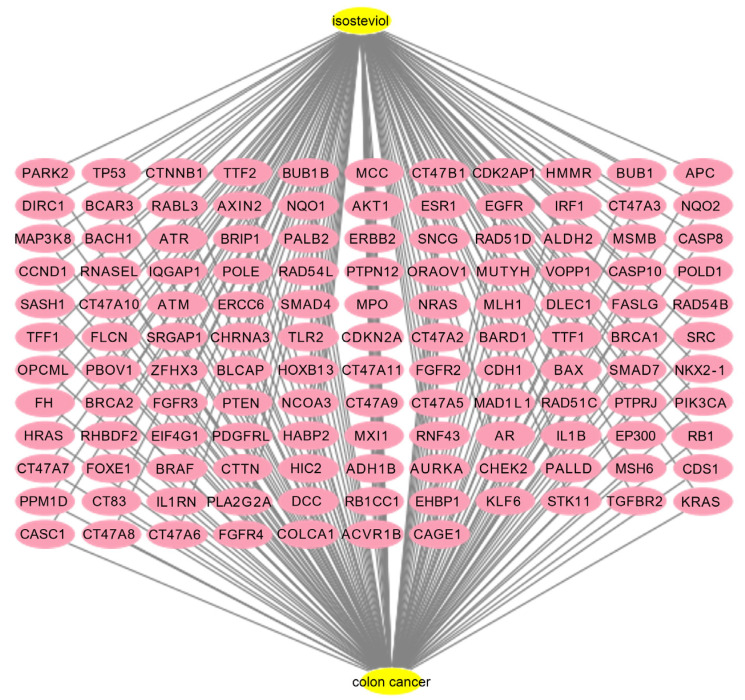
The protein targets most highly involved in interactions with isosteviol and colon cancer.

The Venn diagram in Figure 6 shows 128 common targets between isosteviol and colon cancers. The most highly involved common targets are presented in Figure 7.

#### 3.3.3. PPI Network

**Figure 8:**
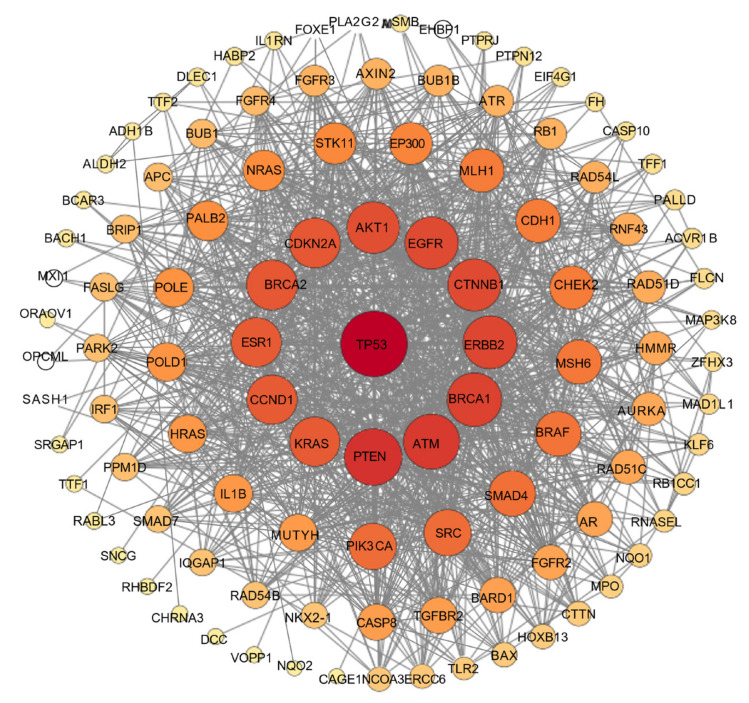
PPI network of the most interconnected targets involved in isosteviol and colon cancer. Node size and color correspond to the degree of interconnection.

The investigation of protein–protein interactions (PPIs) enhances our understanding of the fundamental mechanisms underlying cellular biological processes; meanwhile, it holds significant relevance for disease treatment and drug design. Therefore, identifying PPIs is crucial for understanding the intricate functional interactions found within cells and organisms [27]. A PPI network is used to examine the relationships among the shared targets identified in a recognized compound–target–disease network. This approach provides a comprehensive understanding of the pathological or biological process under investigation. The size and color of the node indicate the level of interaction; specifically, a larger and deeper node signifies a higher degree of interconnection with the target, which correlates with its relevance to disease progression [28]. The results of the PPI network are presented in Figure 8.

#### 3.3.4. Molecular Docking Analysis

**Figure 9:**
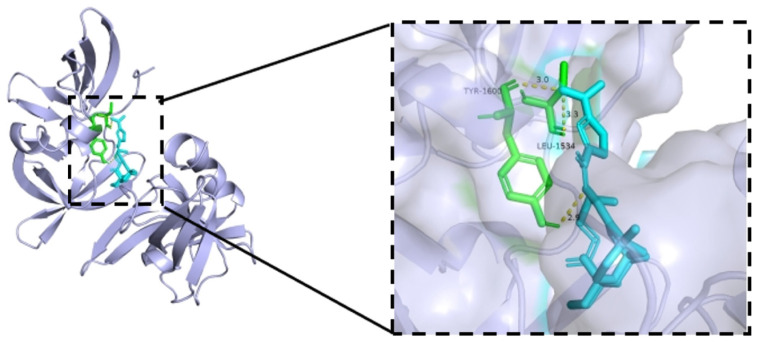
The binding models of derivative **8** with TP53 (6VIP).

Isosteviol and derivative **8** were intended to trigger apoptosis by activating TP53, which is a frequently detected gene-driven gene mutation [29]. To validate this hypothesis, a docking simulation was conducted to place derivative **8** into the active site of TP53 (6VIP) [30] to determine potential binding models (Figure 9).

As shown in Figure 9, derivative **8** interacted with the targeted protein TP53 (6VIP), forming three hydrogen bonds. These three hydrogen bonds involved a nitrogen atom on the nitro group and a nitrogen atom in the Schiff base structure, and generated a docking score of –11.84 Kcal/mol. The nitrogen atom on the nitro group was located 3.0 Å away from the protein residue Tyr-1600 and 3.3 Å away from the protein residue Leu-1534. The nitrogen atom in the Schiff base structure was located 2.9 Å away from the protein residue Tyr-1600. In summary, derivative **8** bound well to protein TP53 and possesses the potential to be a new TP53 protein activator.

## 4. Conclusions

In summary, isosteviol derivatives (**2**–**22**) were synthesized and their antiproliferative activities were also evaluated. The results showed that derivatives **2**, **8**–**9**, **11**–**12,** and **22** possessed moderate inhibitory potency on HCT116 cells. Among them, derivatives **2** and **8** presented remarkable inhibitory potency with IC_50_ values of 7.20 and 6.31 μM, respectively. Notably, derivative **8** exhibited a remarkable inhibitory effect on HCT116 (6.20 μM) and HepG2 (39.84 μM). Based on the bioevaluation data (Table 2), the SARs of the isosteviol derivatives are shown in Figure 3. Cell cycle analysis indicated that derivative **8** induced a dose-dependent accumulation of cells in the G1 phase (G1 phase arrest). The annexin V-APC/PI apoptosis assay revealed that treating HCT116 cells with derivative **8** increased the percentage of apoptotic cells. Molecular docking predicted the binding mode and provided possible explanations for the high inhibitory activity. The results suggested that derivative **8** might be further developed as a high-performance anticancer agent. In addition, in-depth investigation on the pharmacokinetic features, apoptosis pathways, and cancer-targeting formulations of derivative **8** is going to be carried out in our laboratory.

## Figures and Tables

**Table 1:** The chemical structures of isosteviol and the derivatives **2**–**13** and **15**–**22**.

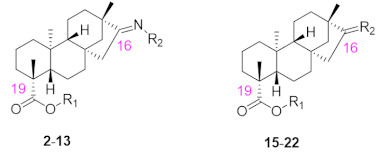				
Compound	R1	R2	IC_50_ (μM) ^1^	
			HCT116	HepG2
**Isosteviol**	H	O	24.8 ± 2.43	>100
**2**	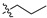	NOH	7.31 ± 1.12	>100
**3**	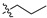	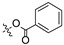	>30	74.43 ± 0.95
**4**	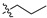	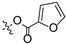	22.75 ± 0.86	>100
**5**	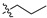	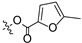	15.52 ± 0.82	>100
**6**	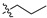	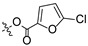	14.47 ± 0.71	>100
**7**	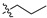	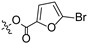	11.97 ± 0.91	>100
**8**	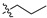	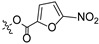	6.20 ± 0.61	39.84 ± 0.43
**9**	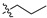	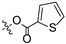	10.62 ± 1.21	74.25 ± 1.53
**10**	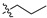	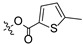	>30	>100
**11**	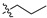	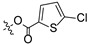	10.44 ± 0.84	>100
**12**	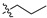	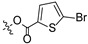	10.00 ± 0.65	>100
**13**	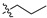	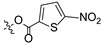	>30	>100
**15**	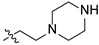	O	>30	>100
**16**	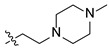	O	>30	>100
**17**	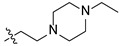	O	>30	>100
**18**	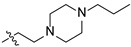	O	21.69 ± 0.35	>100
**19**	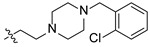	O	28.50 ± 0.16	>100
**20**	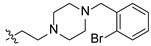	O	23.61 ± 0.63	>100
**21**	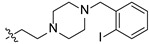	O	15.77 ± 0.69	>100
**22**	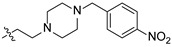	O	10.85 ± 0.26	>100
DOX ^2^			0.16 ± 0.002	0.003 ± 0.004

^1^ Following the CCK-8 methods, cells were incubated with the corresponding compounds for 72 h. IC_50_: concentration that inhibits 50% of cell growth. IC_50_ (μM) values were presented as mean ± SD (*n* = 3). ^2^ DOX was the positive control.

**Table 2:** The apoptosis rate of HCT116 cells induced by different concentrations of derivative **8**.

Derivative 8	Apoptosis (%)			Necrosis (%)
	Early	Late	Total (%)	
NC	44.1	0	44.1	0
3.0 μM	13.0	19.4	32.4	6.83
6.0 μM	12.3	19.7	32.0	8.15
12.0 μM	13.1	41.2	54.3	10.4

## Data Availability

The original contributions presented in this study are included in the article and Supplementary Materials. Further inquiries can be directed to the corresponding author.

## Supplementary Materials

The following supporting information can be downloaded at: https://www.mdpi.com/article/10.3390/biomedicines13040793/s1. Figure S1: ^1^H NMR spectrum of derivative **3**; Figure S2: ^13^C NMR spectrum of derivative **3**; Figure S3: ^1^H NMR spectrum of derivative **4**; Figure S4: ^13^C NMR spectrum of derivative **4**; Figure S5: ^1^H NMR spectrum of derivative **5**; Figure S6: ^13^C NMR spectrum of derivative **5**; Figure S7: ^1^H NMR spectrum of derivative **6**; Figure S8: ^13^C NMR spectrum of derivative **6**; Figure S9: ^1^H NMR spectrum of derivative **7**; Figure S10: ^13^C NMR spectrum of derivative **7**; Figure S11: ^1^H NMR spectrum of derivative **8**; Figure S12: ^13^C NMR spectrum of derivative **8**; Figure S13: ^1^H NMR spectrum of derivative **9**; Figure S14: ^13^C NMR spectrum of derivative **9**; Figure S15: ^1^H NMR spectrum of derivative **10**; Figure S16: ^13^C NMR spectrum of derivative **10**; Figure S17: ^1^H NMR spectrum of derivative **11**; Figure S18: ^13^C NMR spectrum of derivative **11**; Figure S19: ^1^H NMR spectrum of derivative **12**; Figure S20: ^13^C NMR spectrum of derivative **12**; Figure S21: ^1^H NMR spectrum of derivative **13**; Figure S22: ^13^C NMR spectrum of derivative **13**; Figure S23: ^1^H NMR spectrum of derivative **15**; Figure S24: ^13^C NMR spectrum of derivative **15**; Figure S25: ^1^H NMR spectrum of derivative **16**; Figure S26: ^13^C NMR spectrum of derivative **16**; Figure S27: ^1^H NMR spectrum of derivative **17**; Figure S28: ^13^C NMR spectrum of derivative **17**; Figure S29: ^1^H NMR spectrum of derivative **18**; Figure S30: ^13^C NMR spectrum of derivative **18**; Figure S31: ^1^H NMR spectrum of derivative **19**; Figure S32: ^13^C NMR spectrum of derivative **19**; Figure S33: ^1^H NMR spectrum of derivative **20**; Figure S34: ^13^C NMR spectrum of derivative **20**; Figure S35: ^1^H NMR spectrum of derivative **21**; Figure S36: ^13^C NMR spectrum of derivative **21**; Figure S37: ^1^H NMR spectrum of derivative **22**; Figure S38: ^13^C NMR spectrum of derivative **22**.
