# Supplementary material for: Synthesis and Evaluation of Isosteviol Derivatives: Promising Anticancer Therapies for Colon Cancer
Source: Biomedicines. 2025 Mar 25;13(4):793. doi: 10.3390/biomedicines13040793 (PMC12024579; doi:10.3390/biomedicines13040793)
Supplement: Supplementary file 1 [file biomedicines-13-00793-s001.zip › biomedicines-3501675-supplementary.pdf]

biomedicines

Supplementary Material

Synthesis and Evaluation of Isosteviol Derivatives: Promising Anticancer Therapies for Colon Cancer

Yecang Chen [2], Feifei Zhu [1], Yuxin Ding [1], Lin Xing [1], Enxiao Wang [1], Yixiang Fang [1], Ruilong Sheng [4], Qidong Tu [3,*] and Ruihua Guo [1,5,6,*]

1. College of Food Science and Technology, Shanghai Ocean University, Shanghai 201306, China; zff476004403@163.com (F.Z.); dingyuxin0305@163.com (Y.D.); xinglin0220@163.com (L.X.); enxiaowang7374@163.com (E.W.); yxfang2025@163.com (Y.F.)
2. Division of Biomedical Engineering, James Watt School of Engineering, University of Glasgow, Glasgow G12 8LT, UK; 2495282C@student.gla.ac.uk
3. Jiangxi Provincial Key Laboratory of Drug Design and Evaluation, School of Pharmacy, Jiangxi Science & Technology Normal University, Nanchang 330013, China
4. CQM—Centro de Química da Madeira, Campus da Penteada, Universidade da Madeira, 9000-390 Funchal, Portugal; ruilong.sheng@staff.uma.pt
5. Marine Biomedical Science and Technology Innovation Platform of Lin-Gang Special Area, Shanghai 201306, China
6. Department of Marine Biopharmacology, College of Food Science and Technology, Shanghai Ocean University, Shanghai 201306, China

* Correspondence: 1020100994@jxstnu.edu.cn (Q.T.); rhguo@shou.edu.cn (R.G.)

Copies of ^{1}H and ^{13}C NMR Spectra of derivatives 1–25

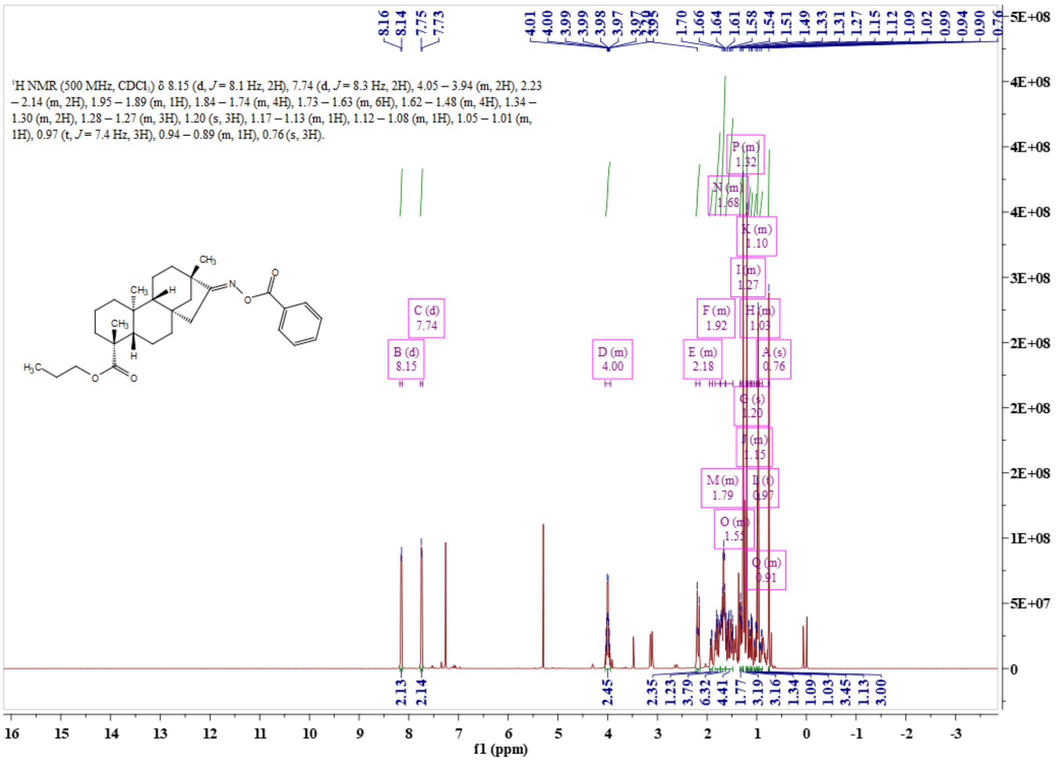

Figure S1. ^{1}H NMR spectrum of derivative **3**.

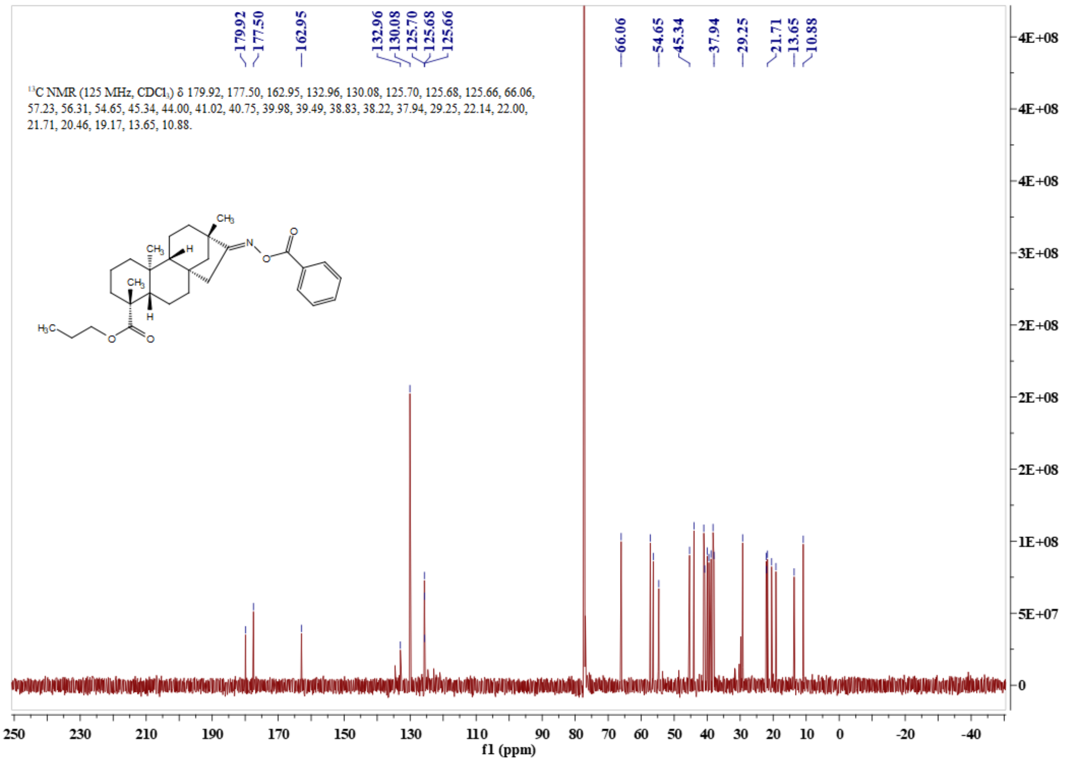

Figure S2. ^{13}C NMR spectrum of derivative **3.**

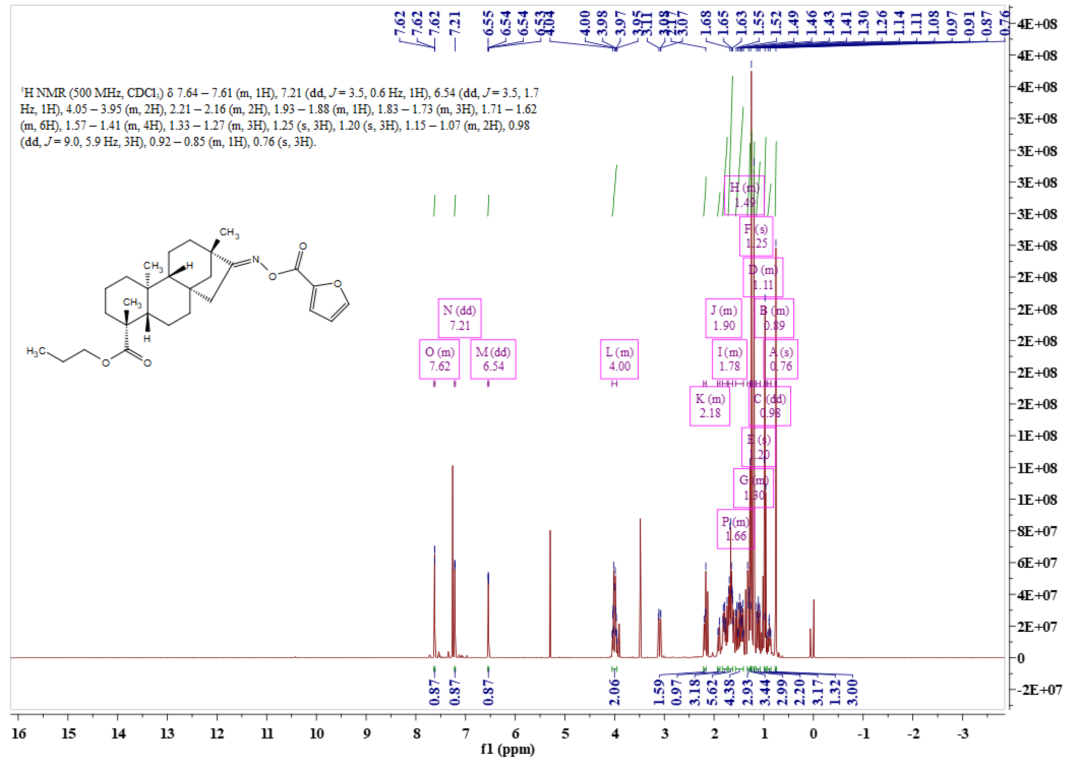

Figure S3. ^{1}H NMR spectrum of derivative **4.**

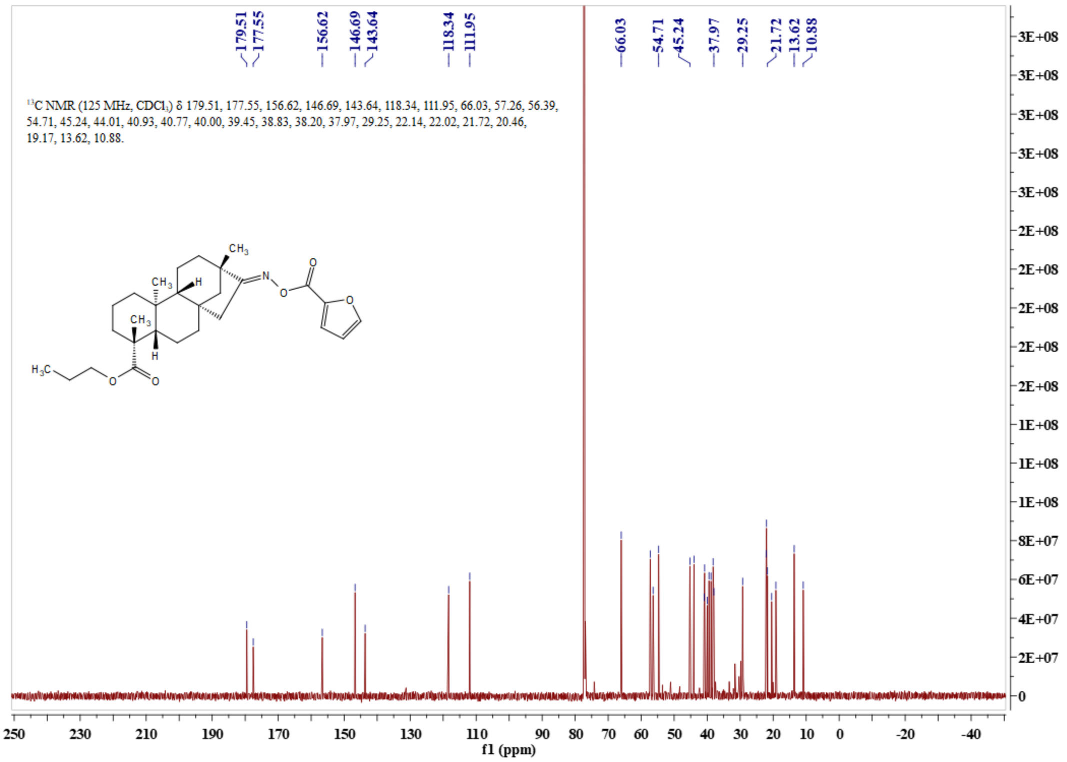

Figure S4. ^{13}C NMR spectrum of derivative **4.**

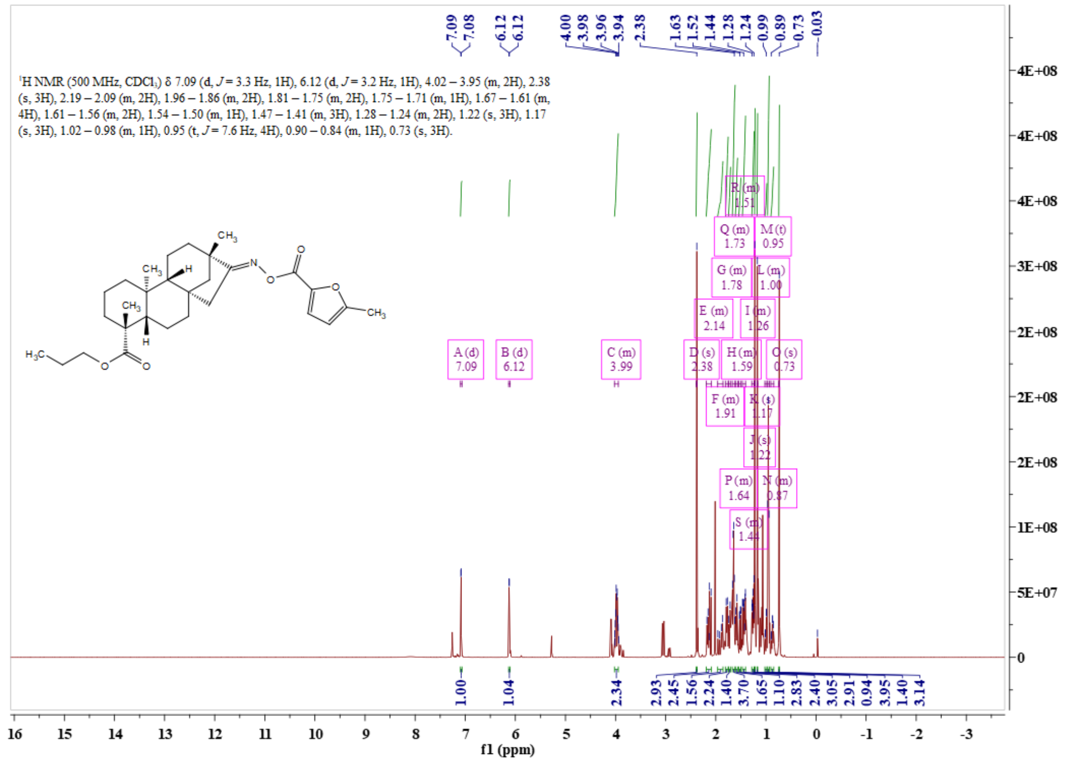

Figure S5. ^{1}H NMR spectrum of derivative **5.**

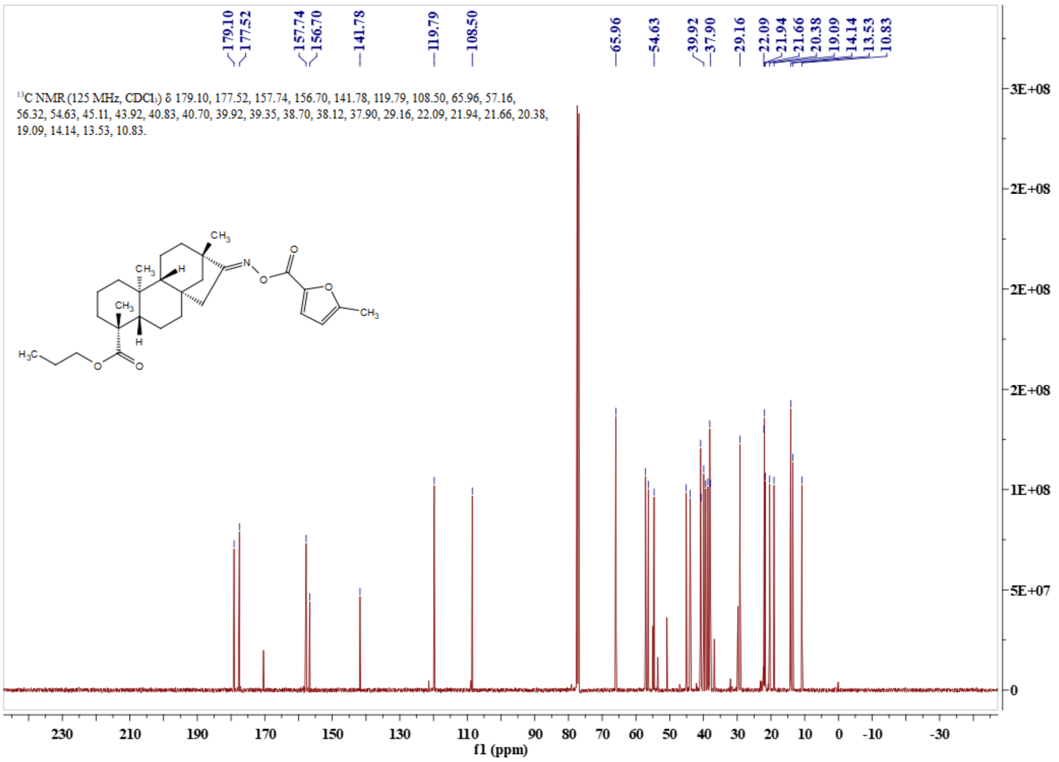

Figure S6. ^{13}C NMR spectrum of derivative **5.**

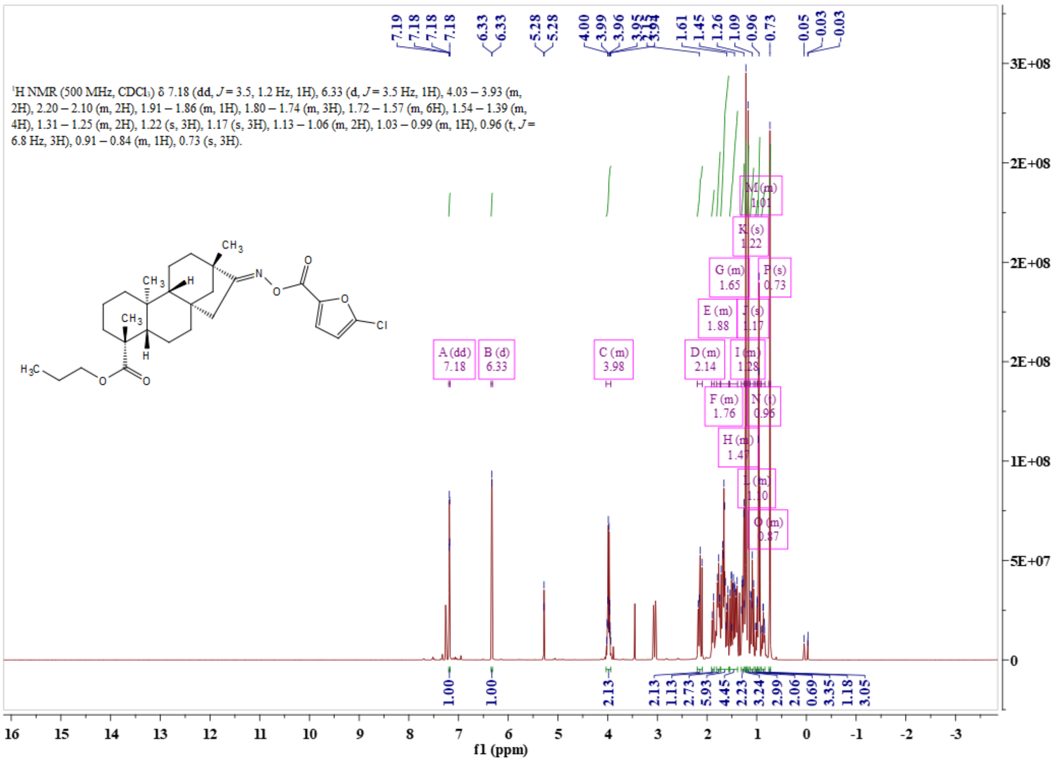

Figure S7. ^{1}H NMR spectrum of derivative **6.**

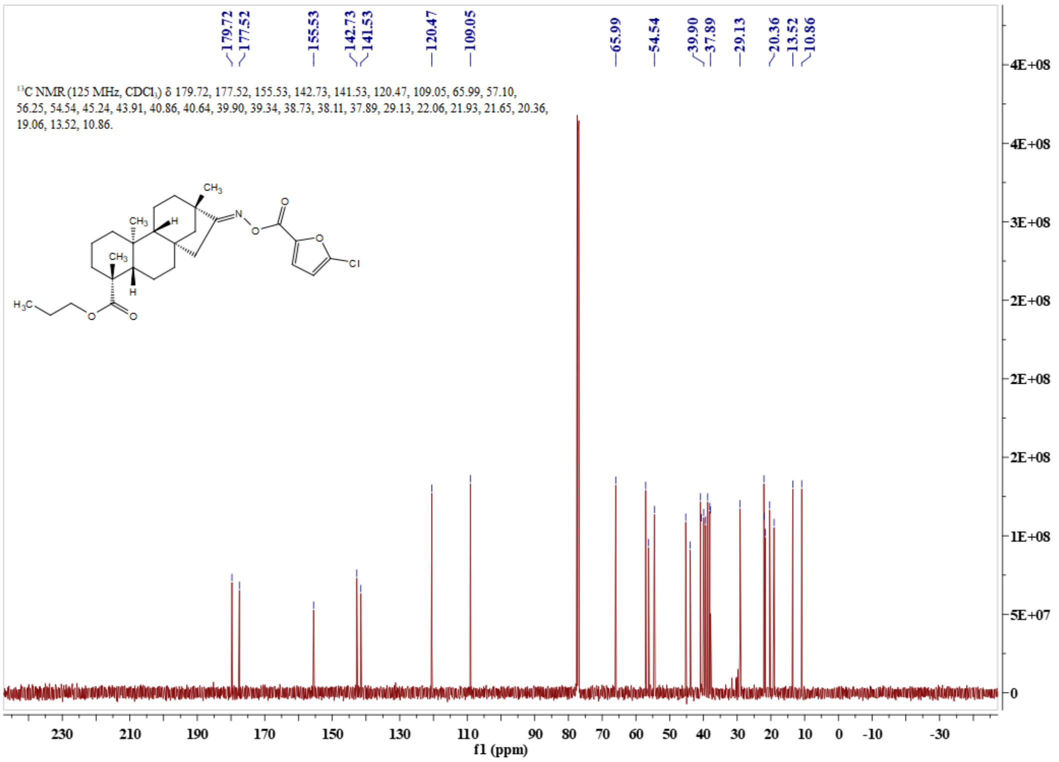

Figure S8. ^{13}C NMR spectrum of derivative **6.**

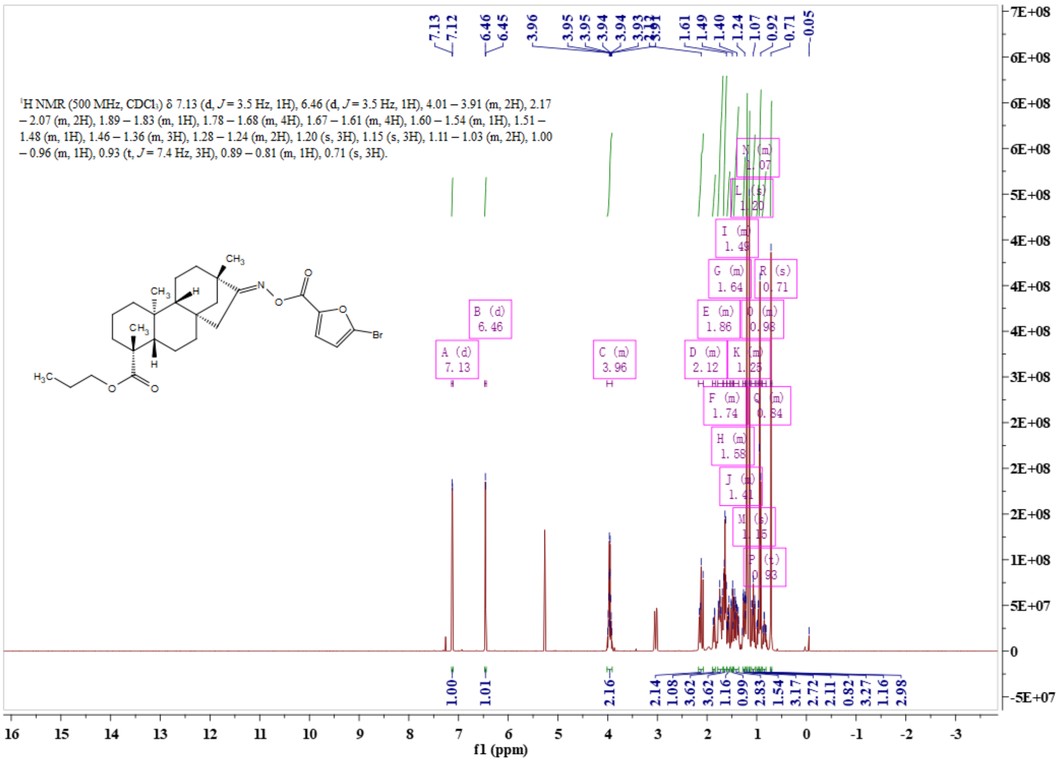

Figure S9. ^{1}H NMR spectrum of derivative **7.**

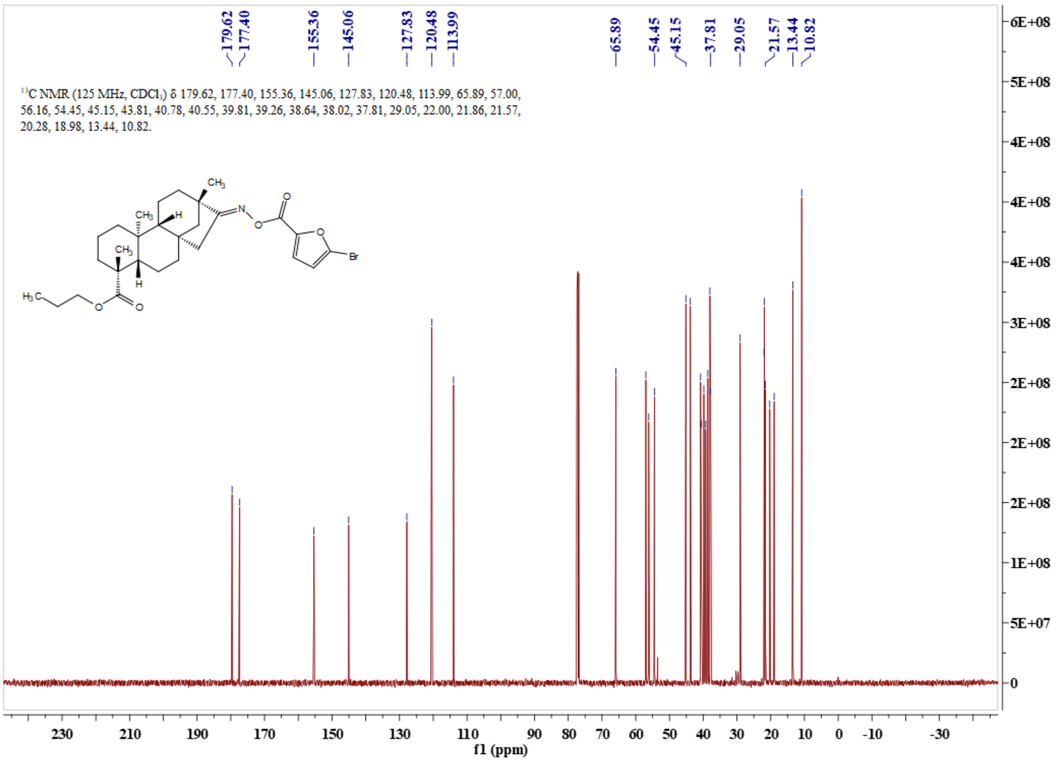

Figure S10. ^{13}C NMR spectrum of derivative **7.**

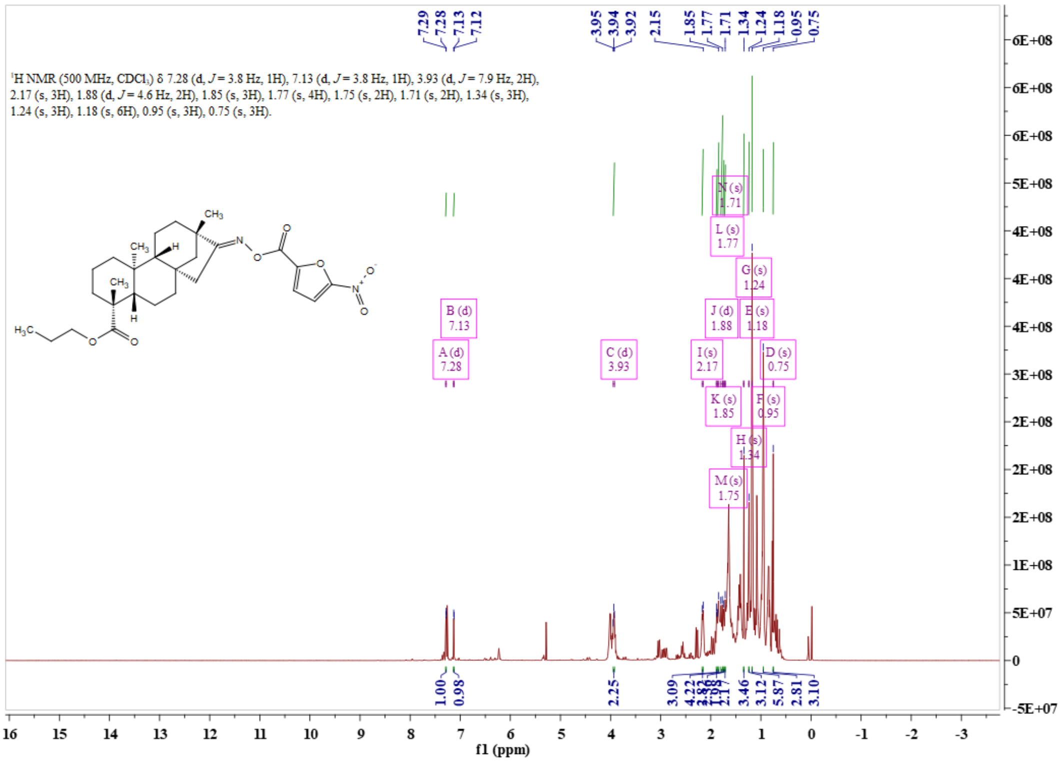

Figure S11. ^{1}H NMR spectrum of derivative **8.**

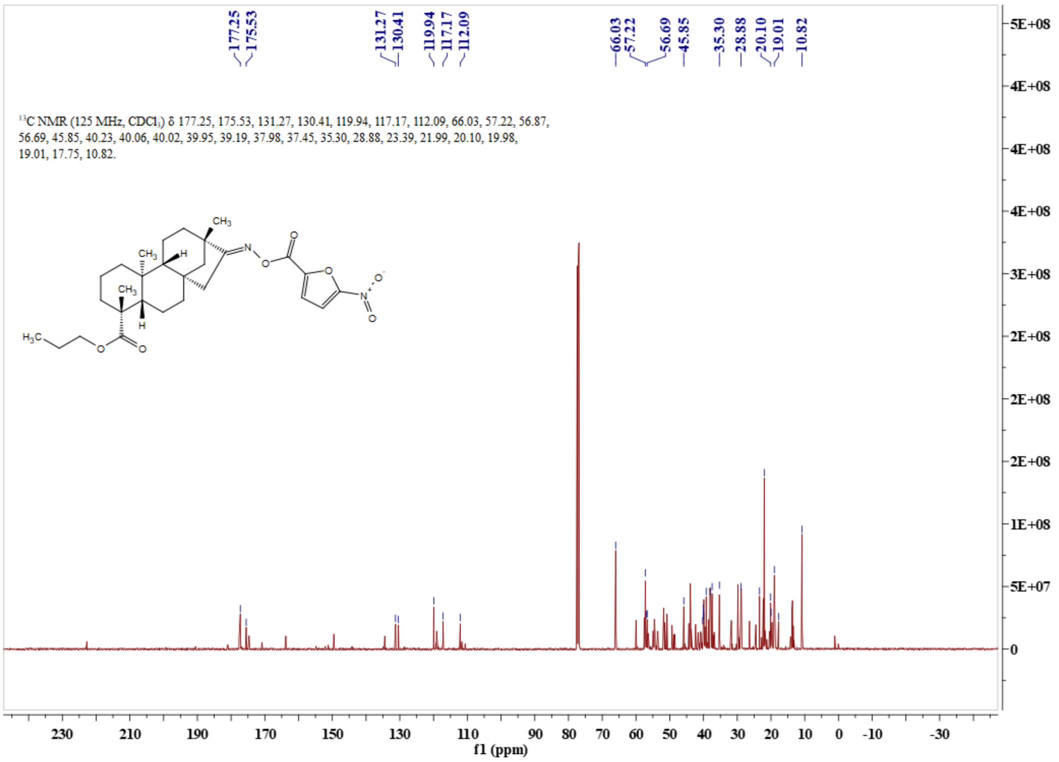

Figure S12. ^{13}C NMR spectrum of derivative **8.**

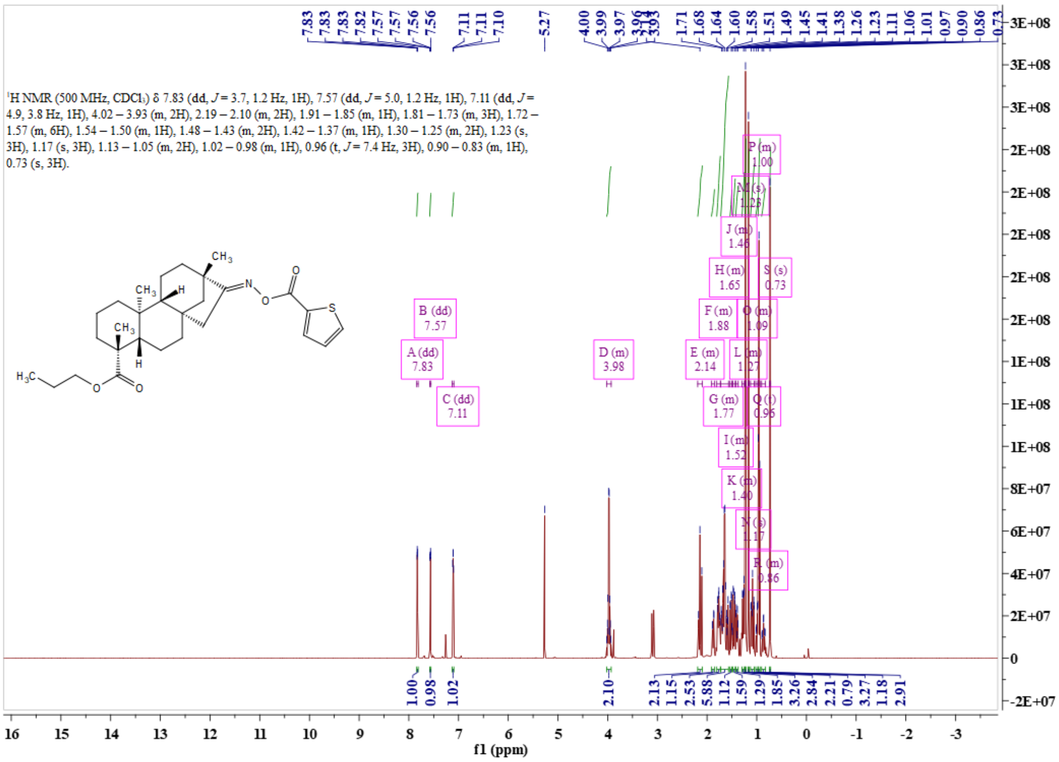

Figure S13. ^{1}H NMR spectrum of derivative **9.**

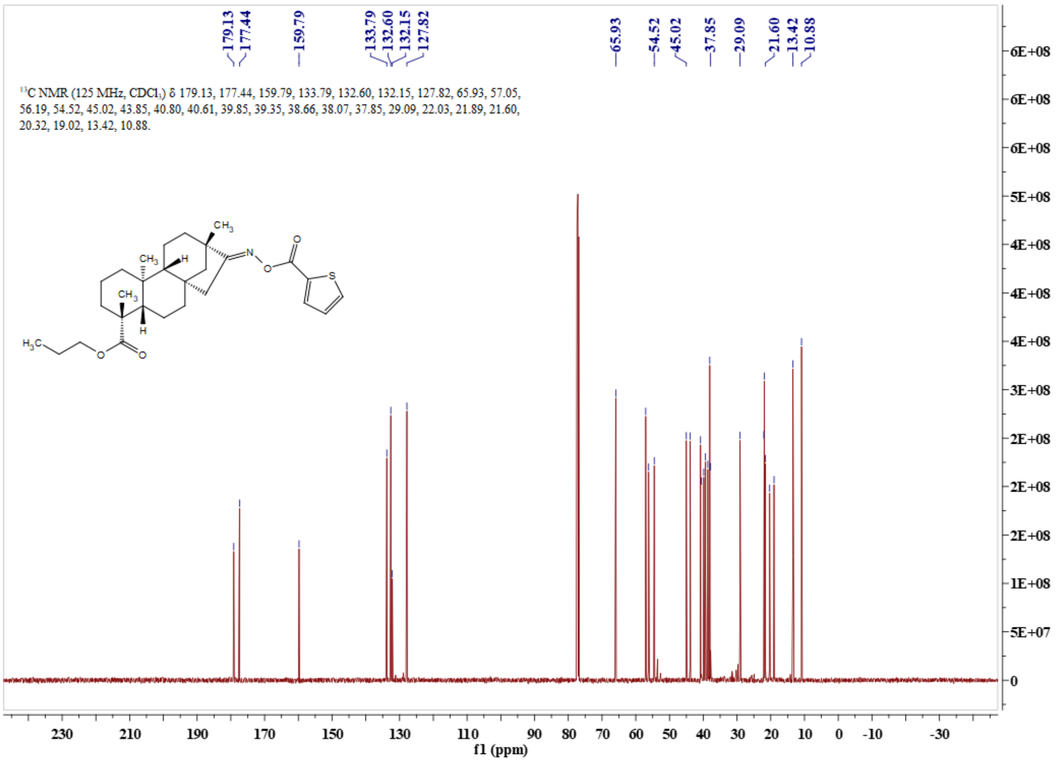

Figure S14. ^{13}C NMR spectrum of derivative **9.**

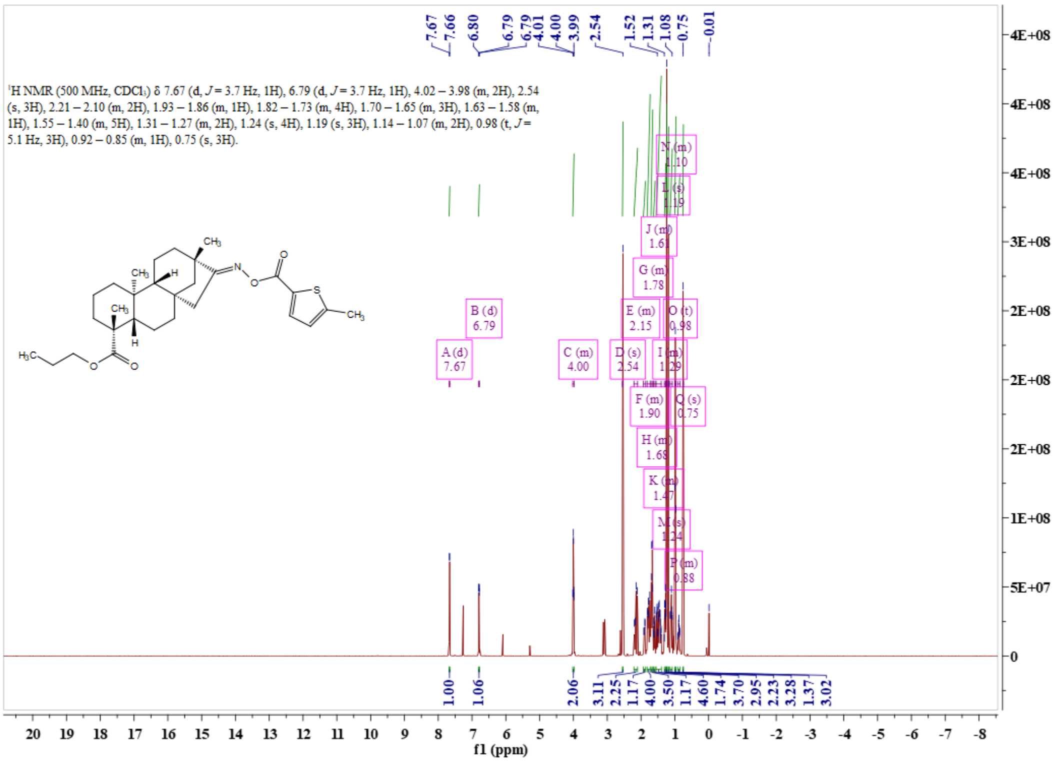

Figure S15. ^{1}H NMR spectrum of derivative **10.**

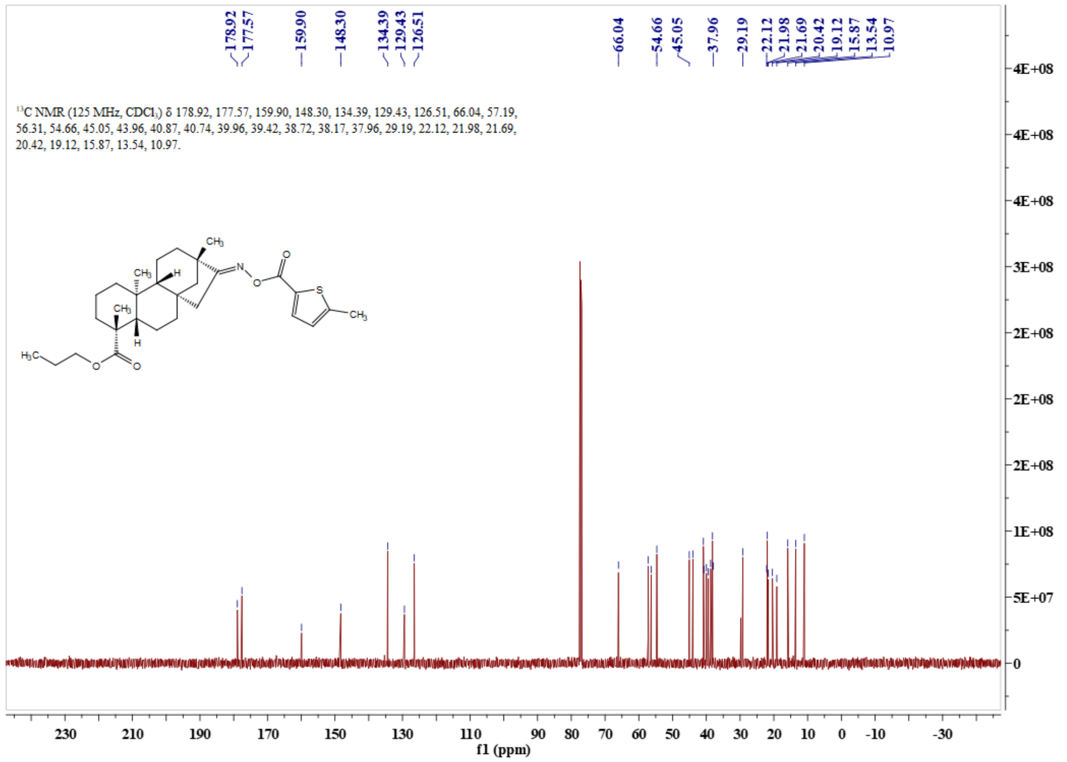

Figure S16. ^{13}C NMR spectrum of derivative **10.**

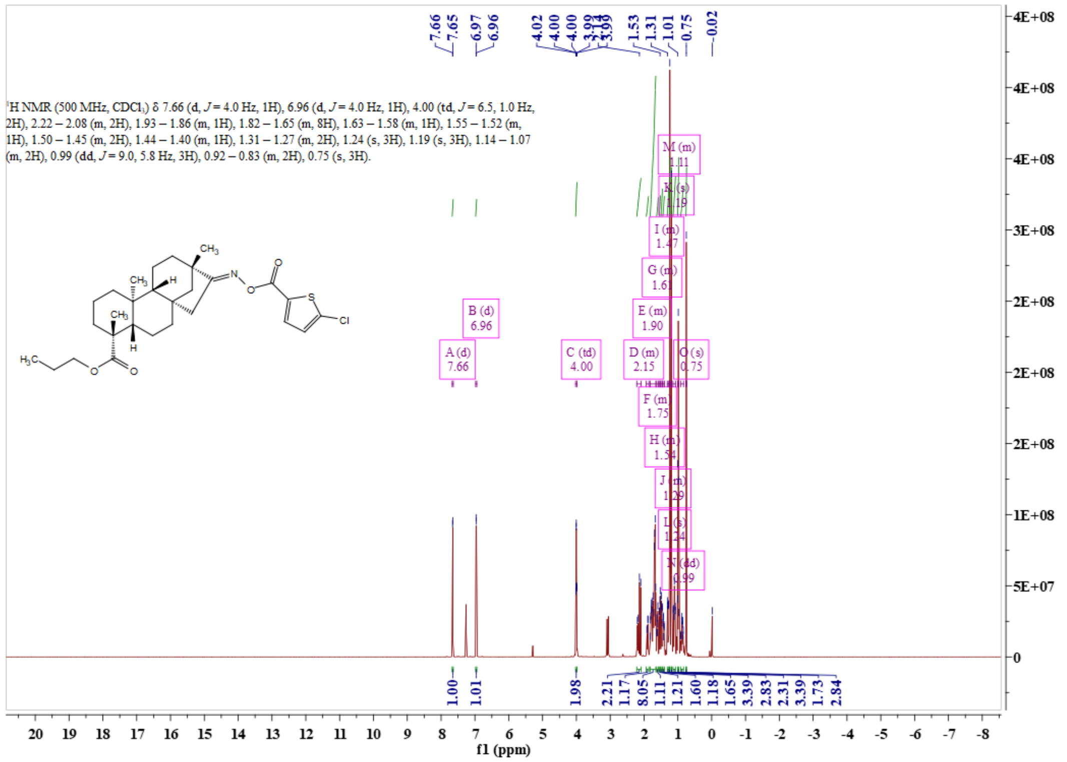

Figure S17. ^{1}H NMR spectrum of derivative **11.**

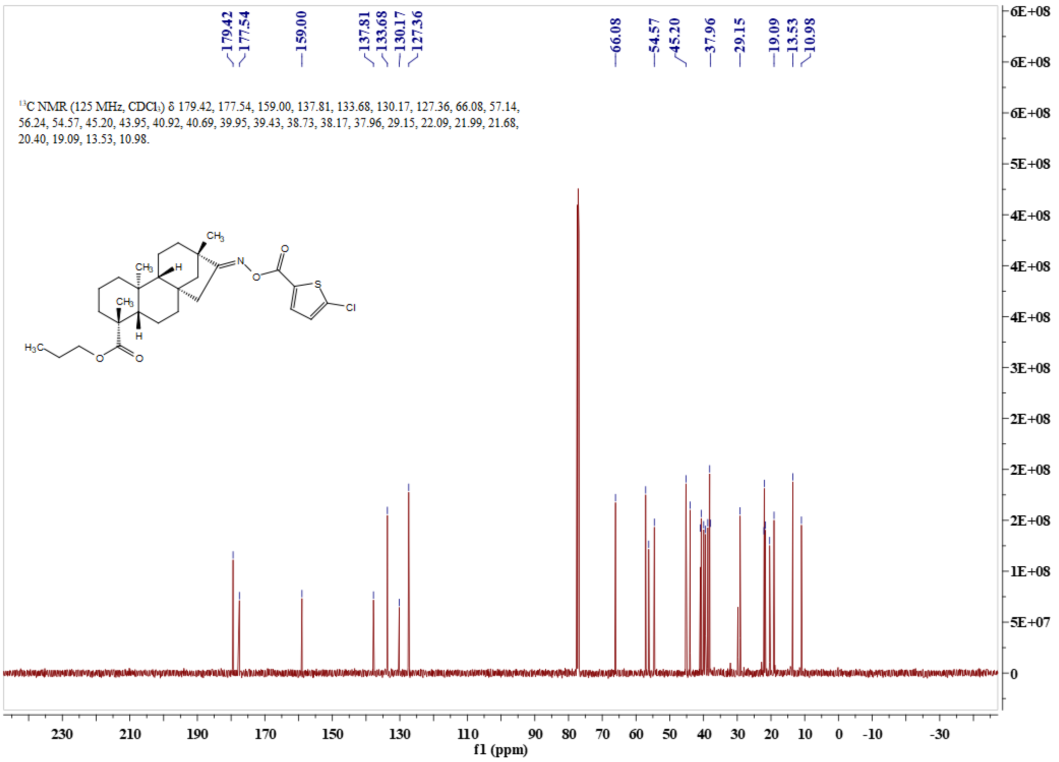

Figure S18. ^{13}C NMR spectrum of derivative **11.**

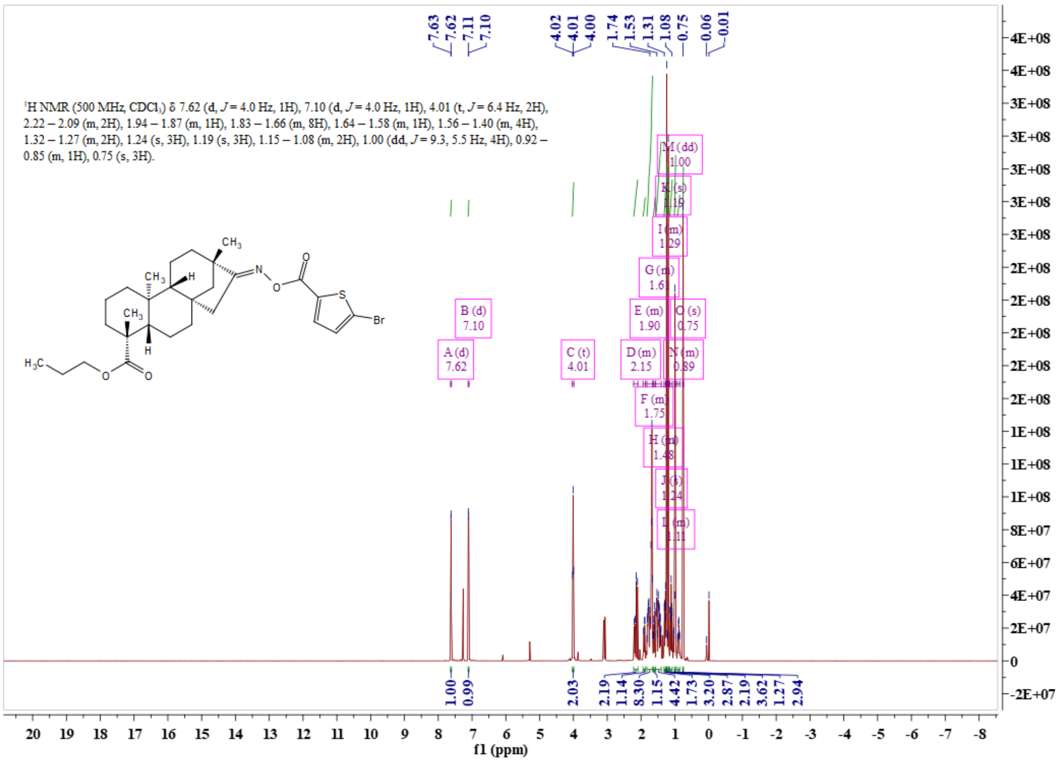

Figure S19. ^{1}H NMR spectrum of derivative **12.**

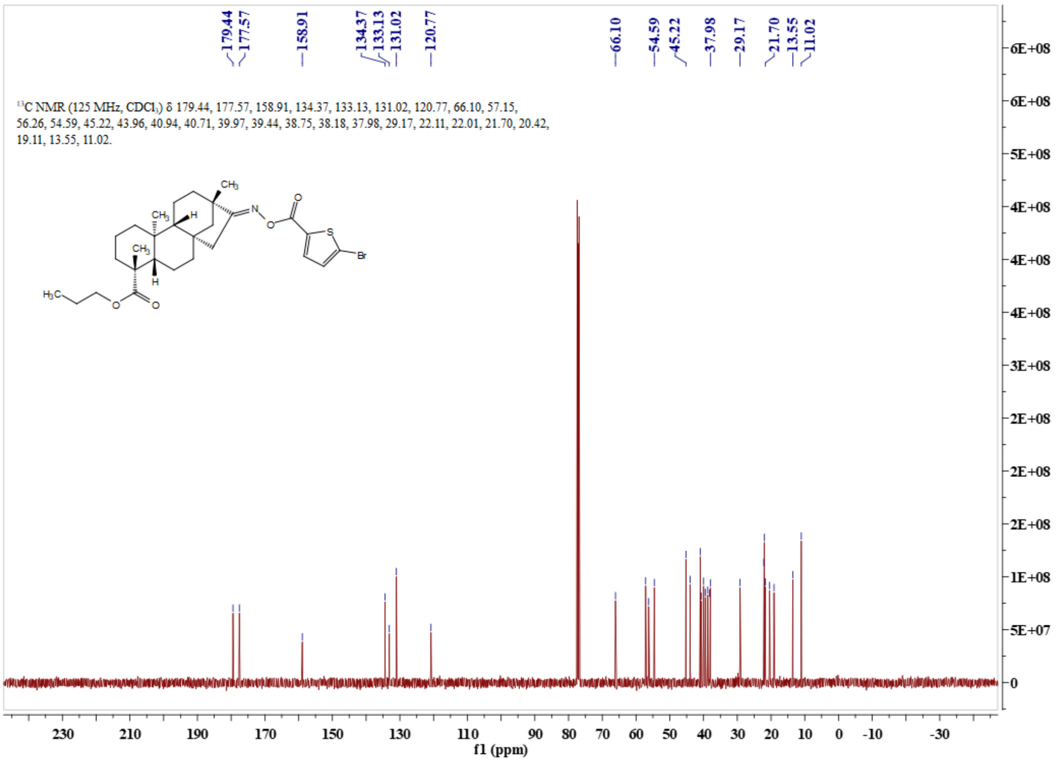

Figure S20. ^{13}C NMR spectrum of derivative **12.**

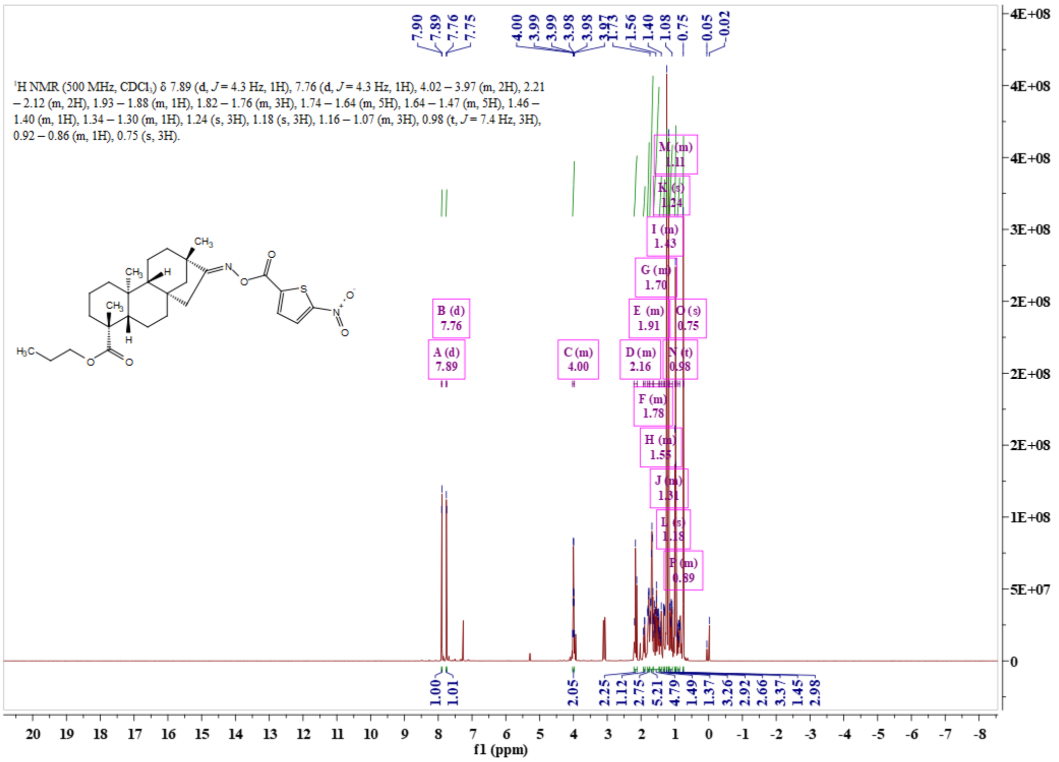

Figure S21. ^{1}H NMR spectrum of derivative **13.**

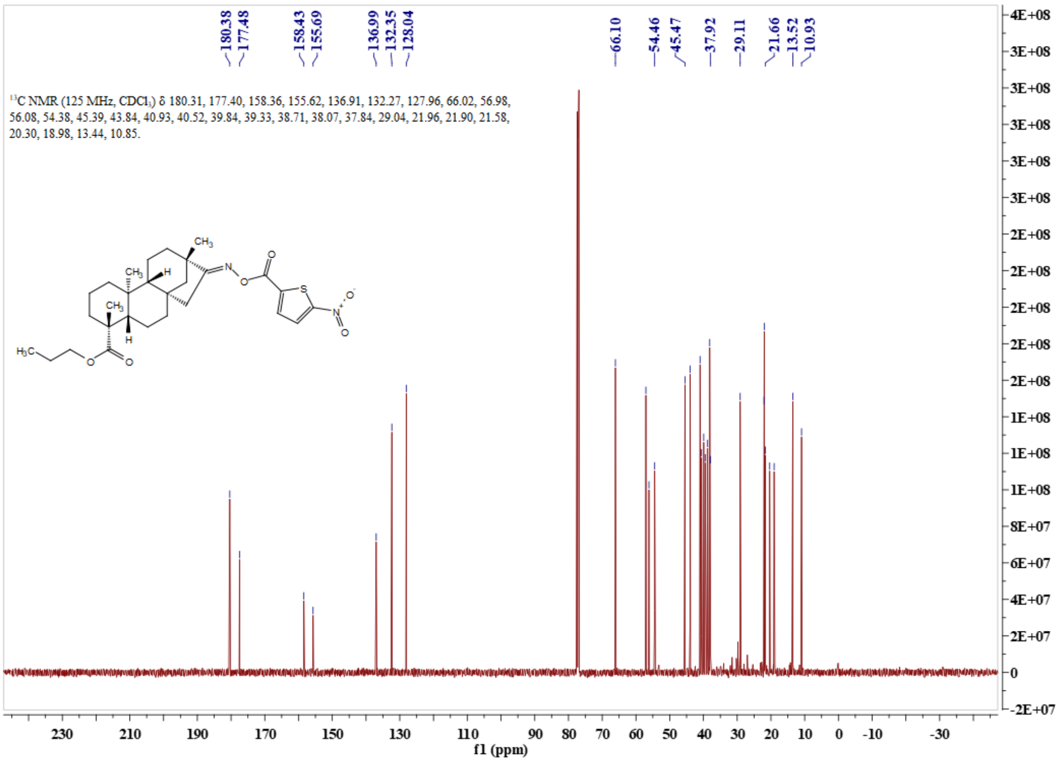

Figure S22. ^{13}C NMR spectrum of derivative **13.**

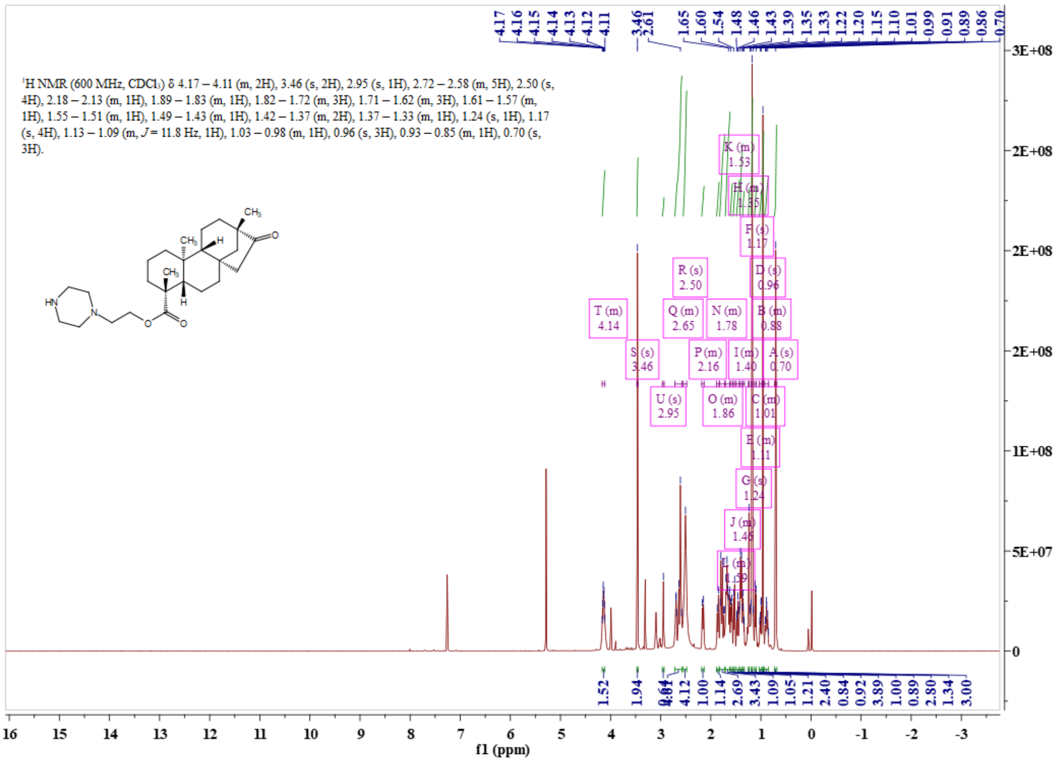

Figure S23. ^{1}H NMR spectrum of derivative **15.**

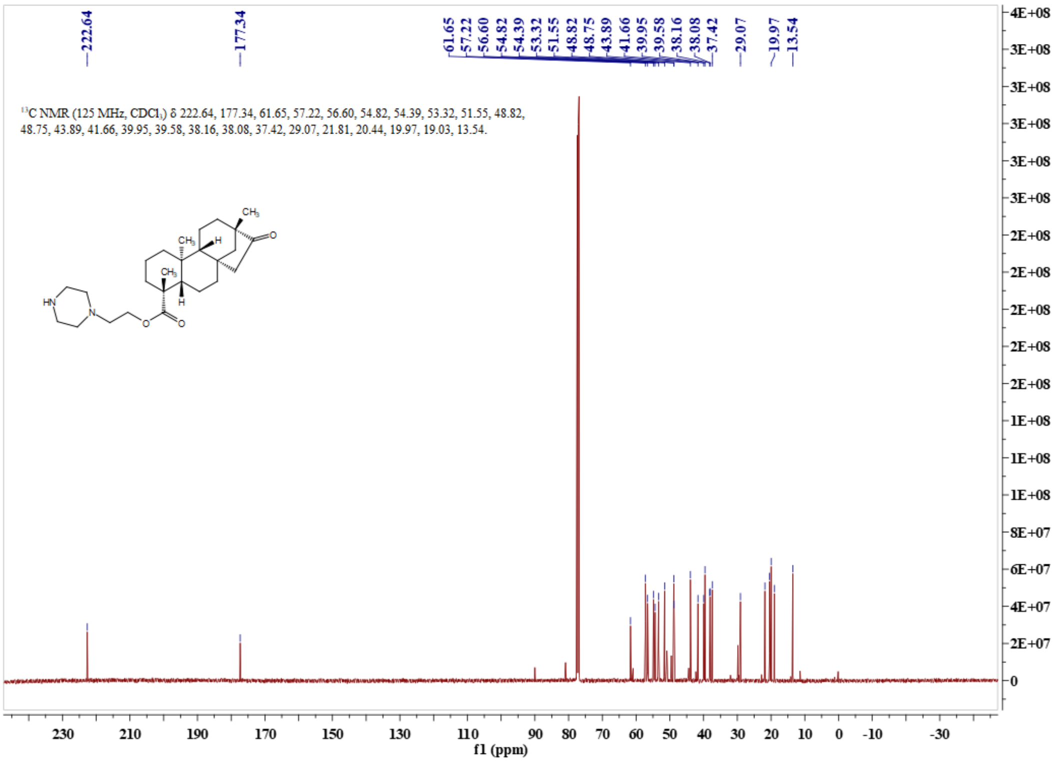

Figure S24. ^{13}C NMR spectrum of derivative **15.**

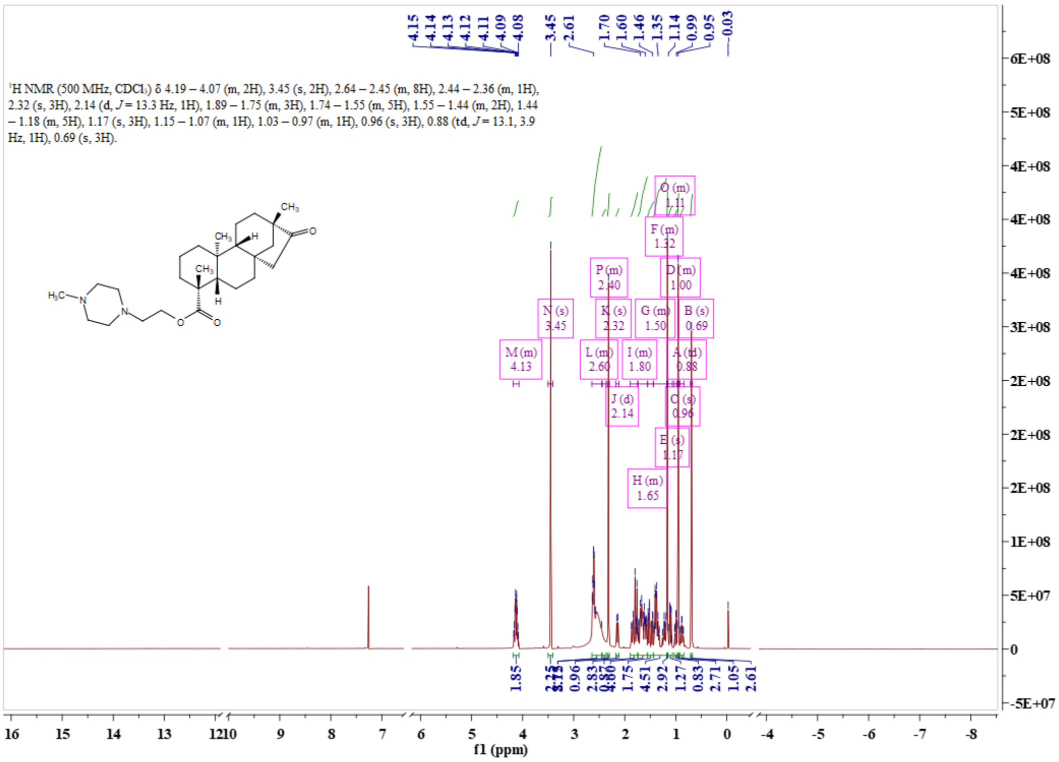

Figure S25. ^{1}H NMR spectrum of derivative **16.**

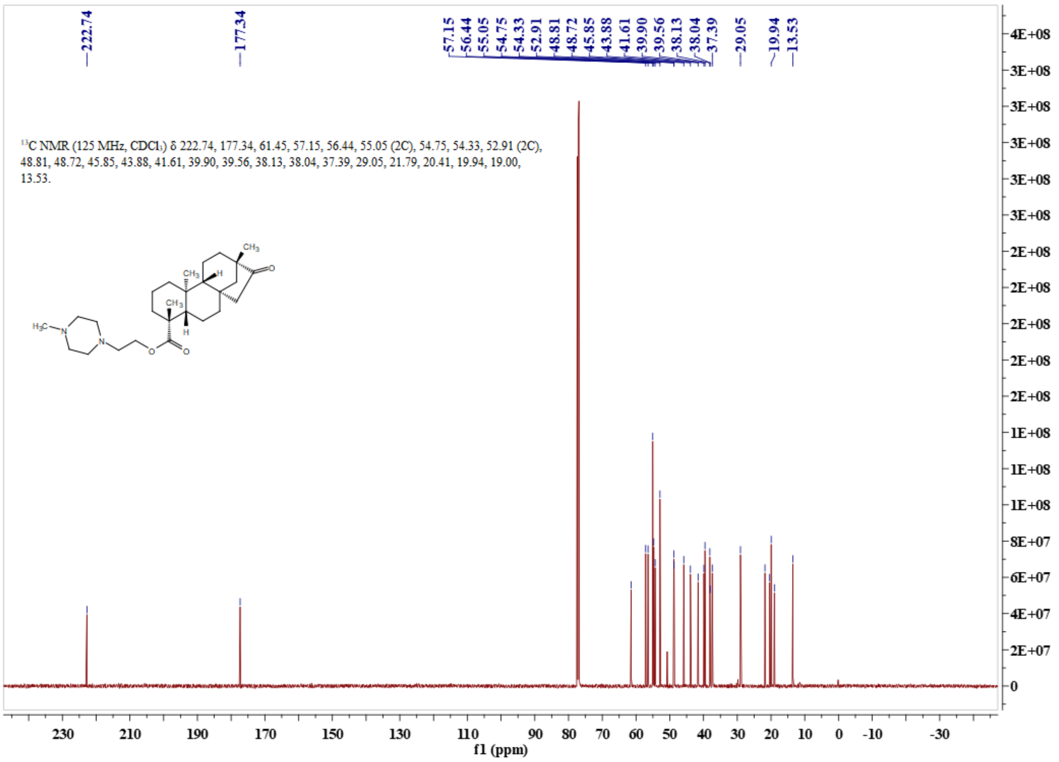

Figure S26. ^{13}C NMR spectrum of derivative **16.**

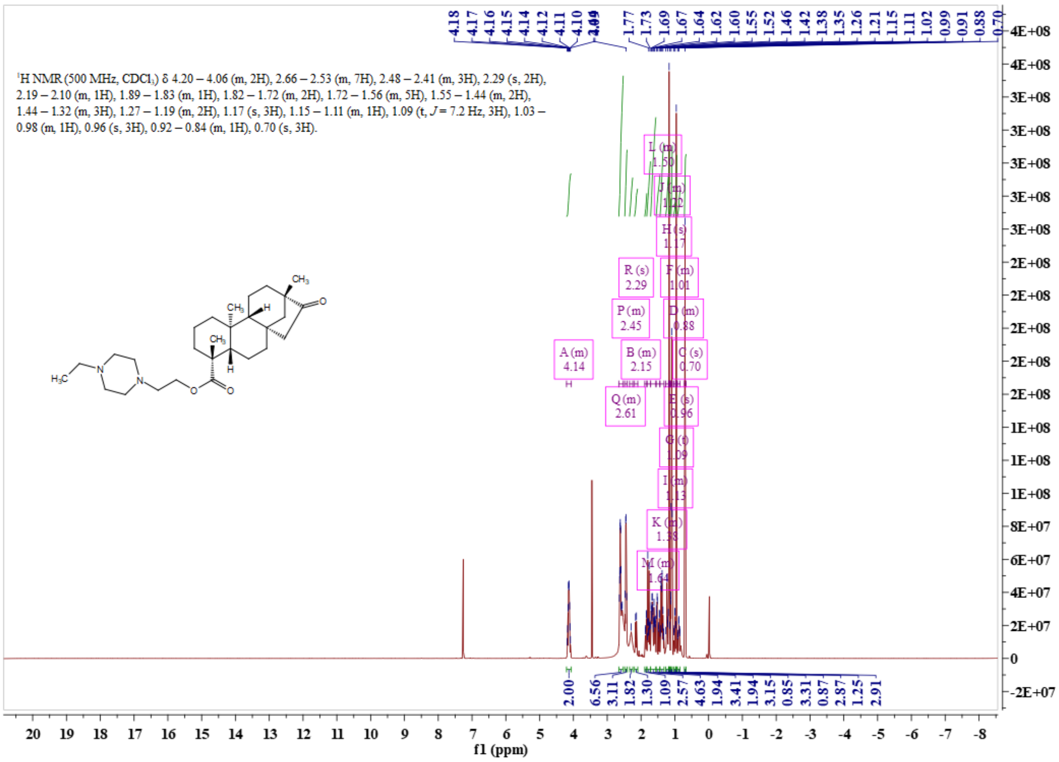

Figure S27. ^{1}H NMR spectrum of derivative **17.**

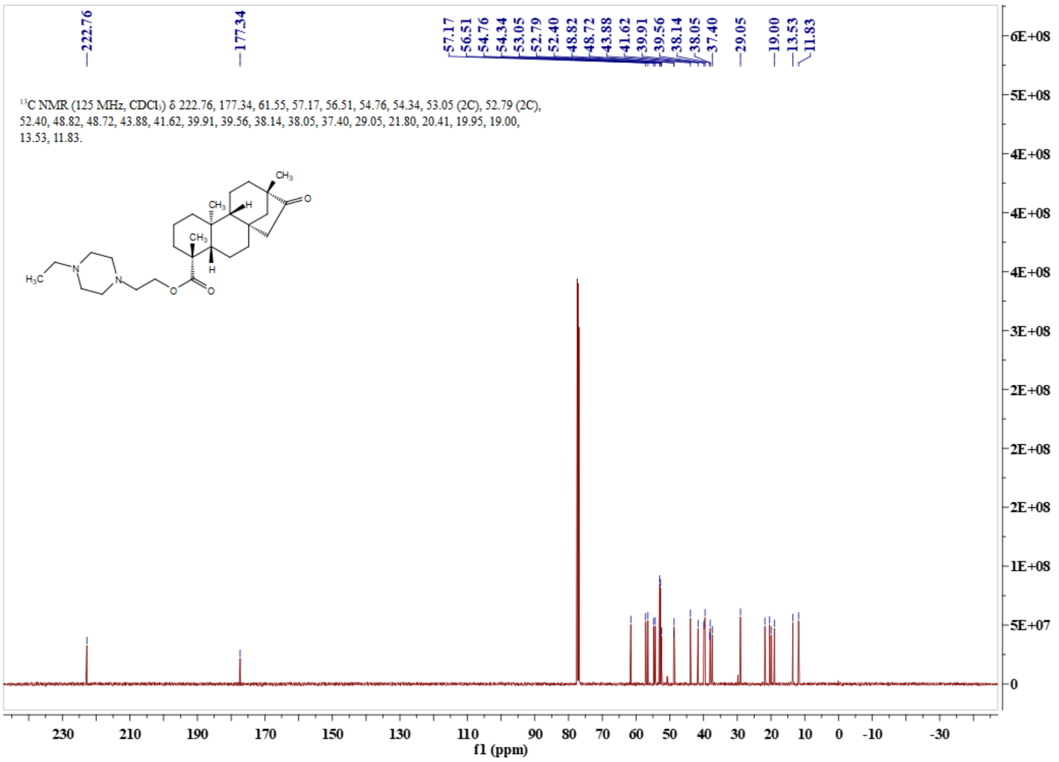

Figure S28. ^{13}C NMR spectrum of derivative **17.**

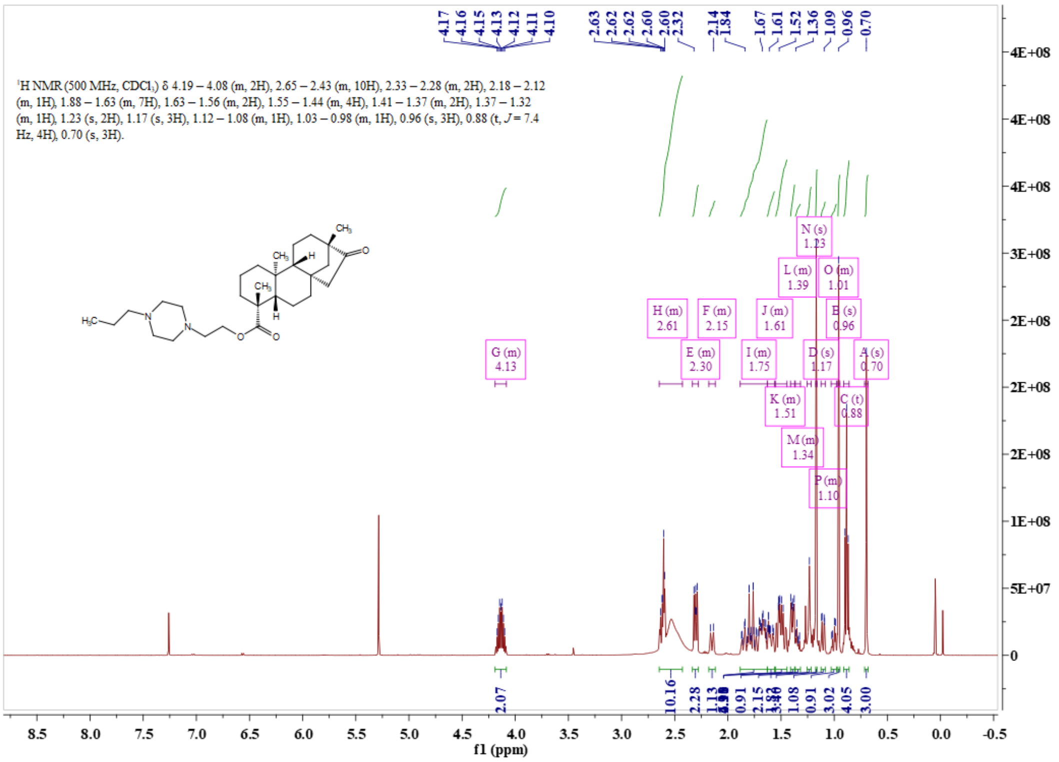

Figure S29. ^{1}H NMR spectrum of derivative **18.**

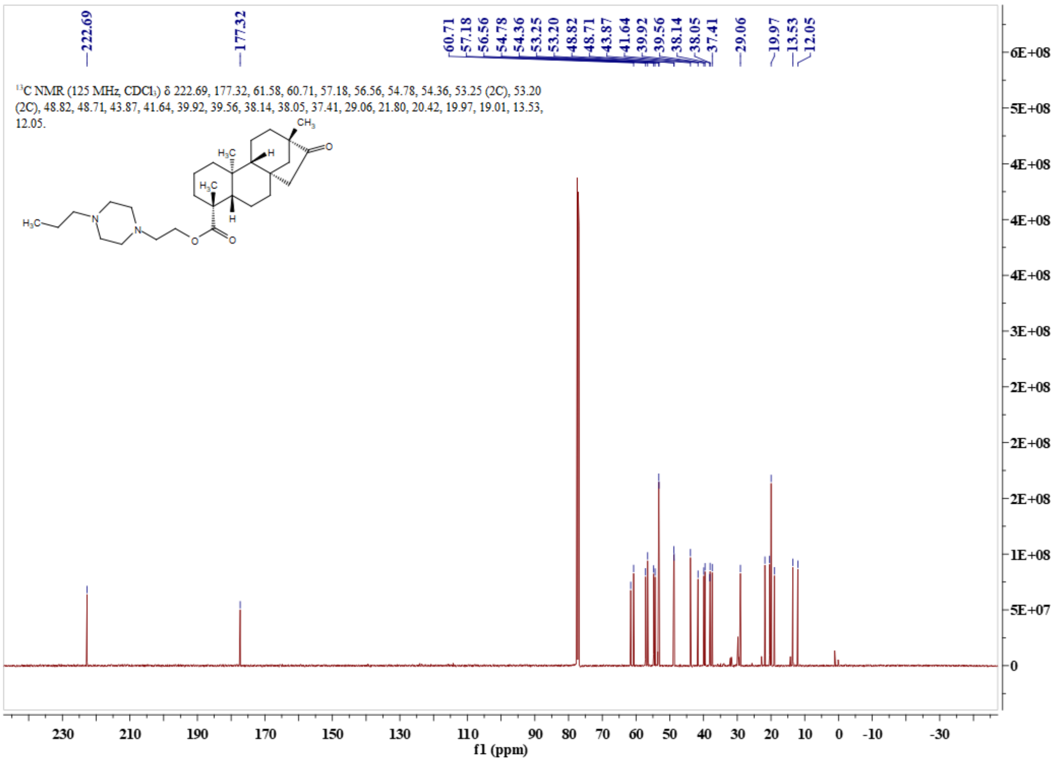

Figure S30. ^{13}C NMR spectrum of derivative **18.**

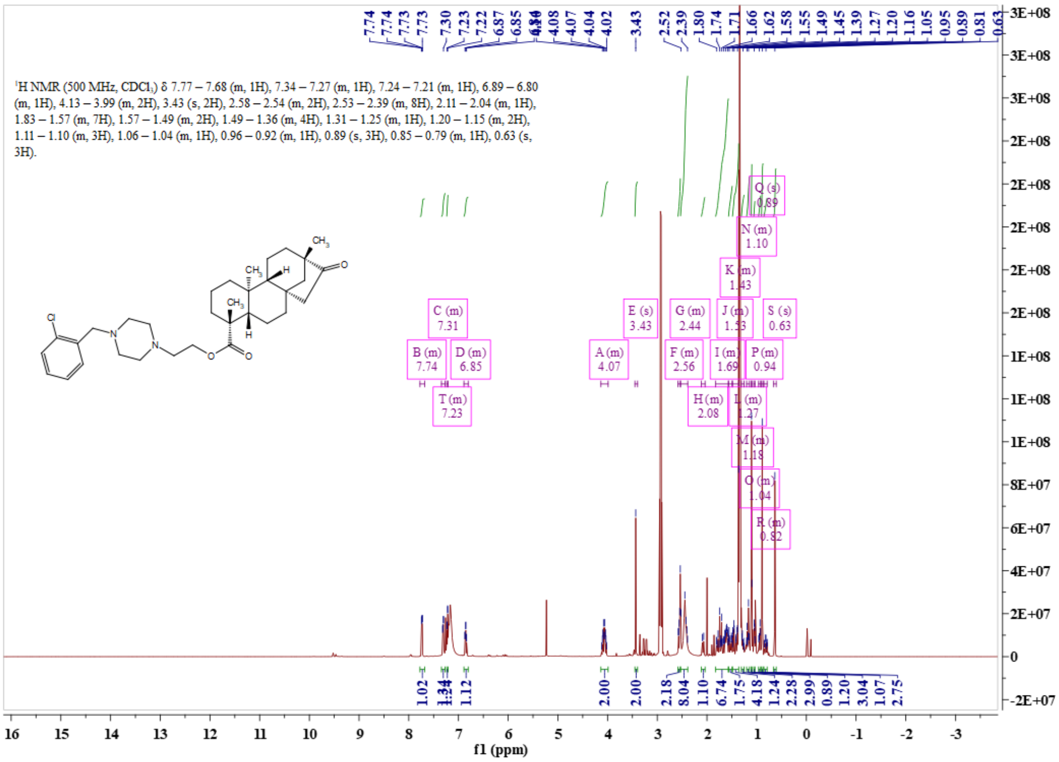

Figure S31. ^{13}C NMR spectrum of derivative **19.**

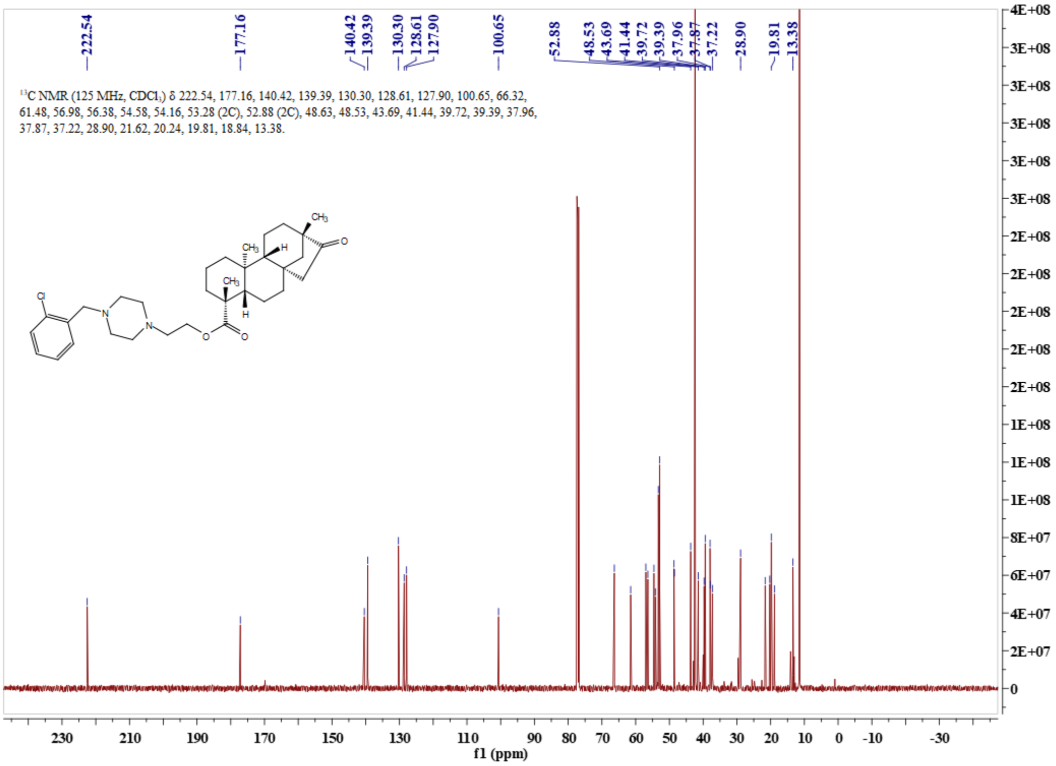

Figure S32. ^{13}C NMR spectrum of derivative **19.**

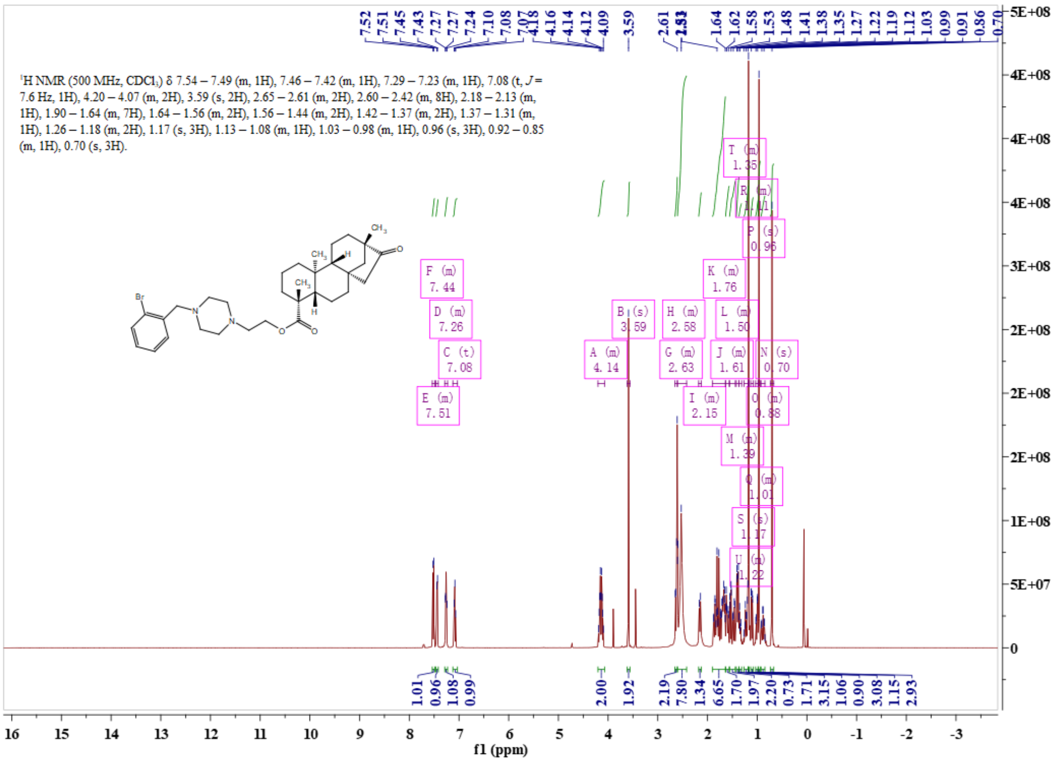

Figure S33. ^{1}H NMR spectrum of derivative **20.**

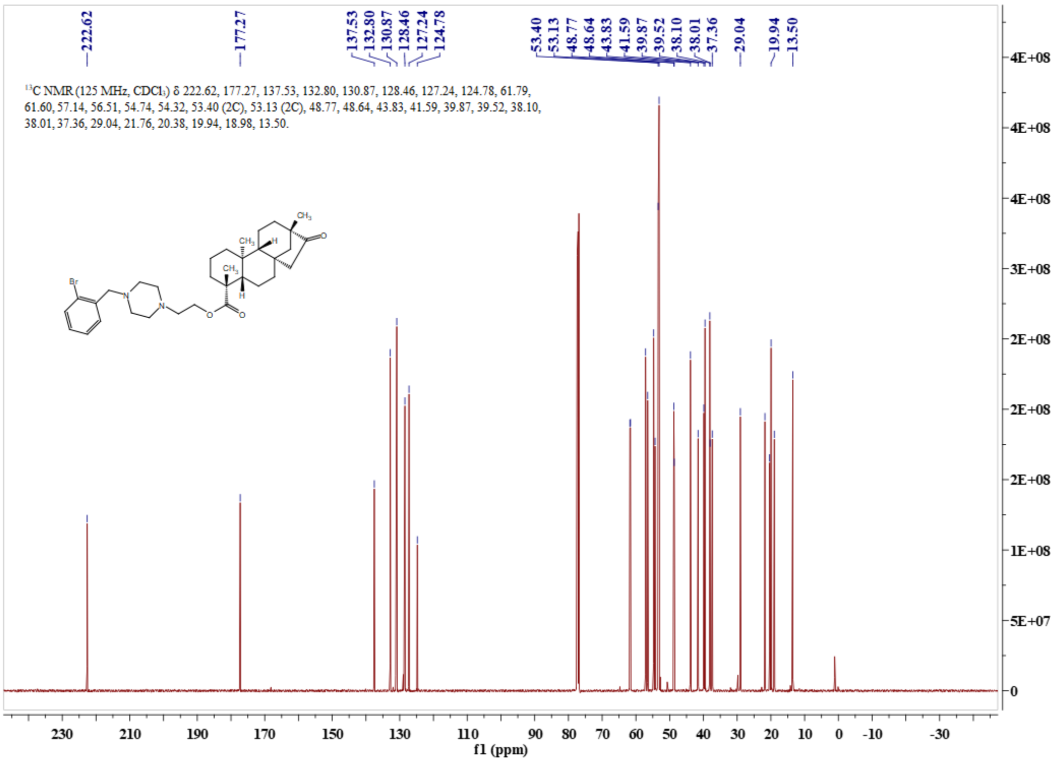

Figure S34. ^{13}C NMR spectrum of derivative **20.**

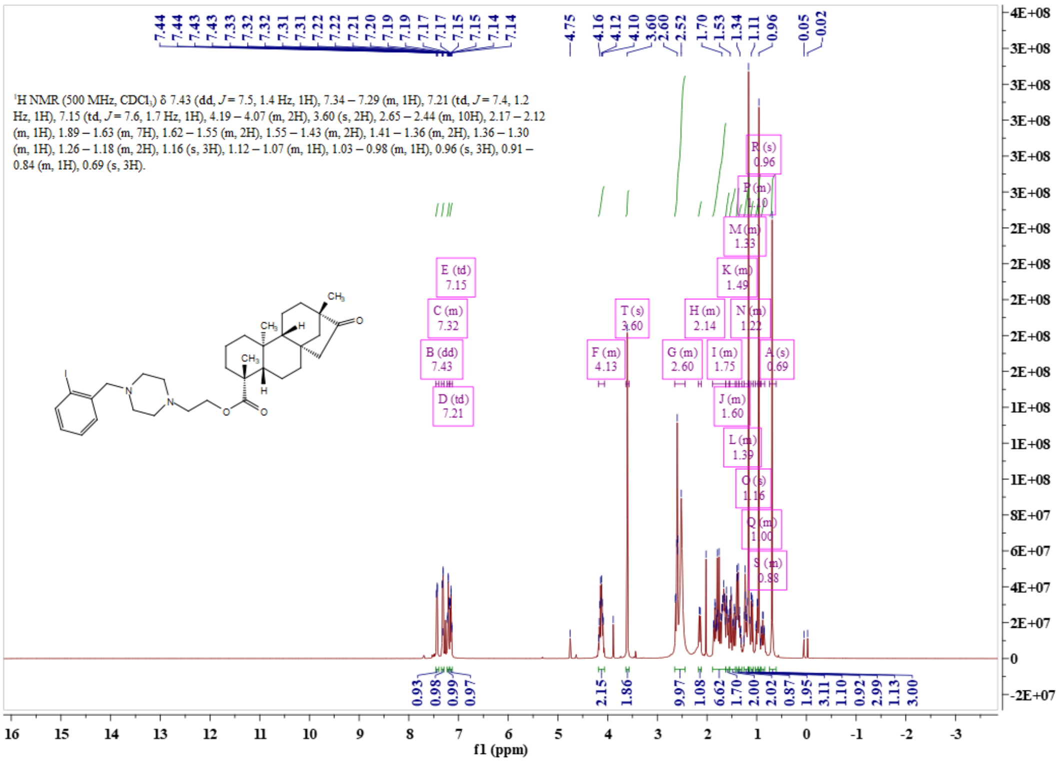

Figure S35. ^{1}H NMR spectrum of derivative **21.**

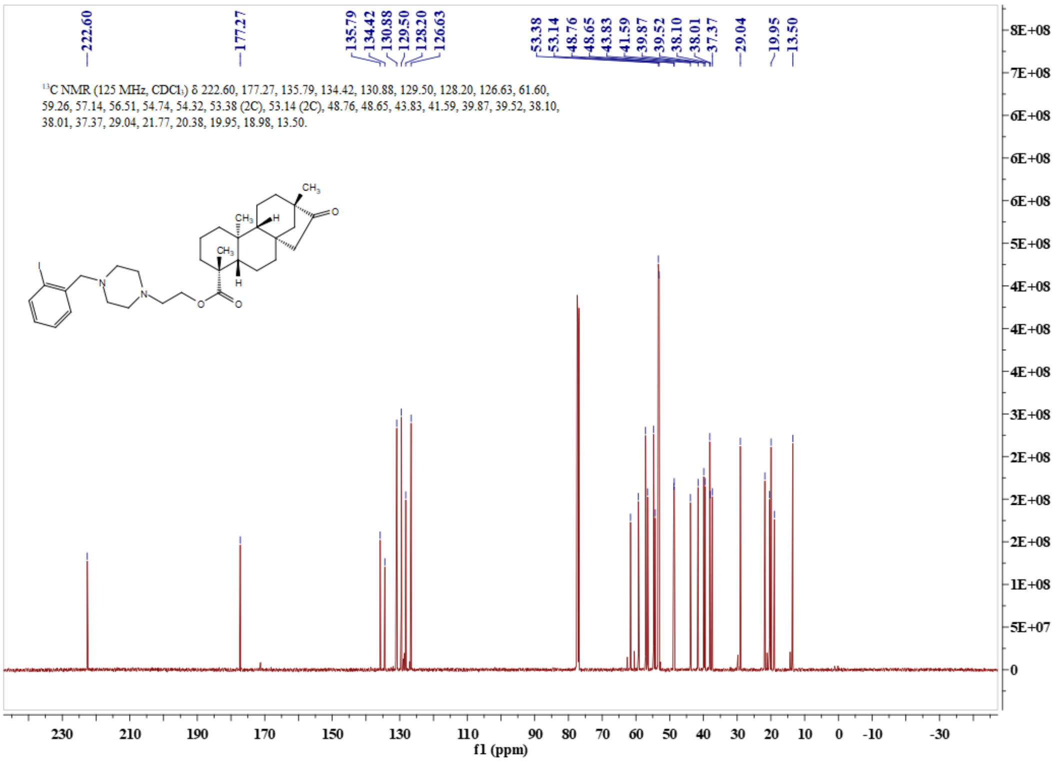

Figure S36. ^{13}C NMR spectrum of derivative **21.**

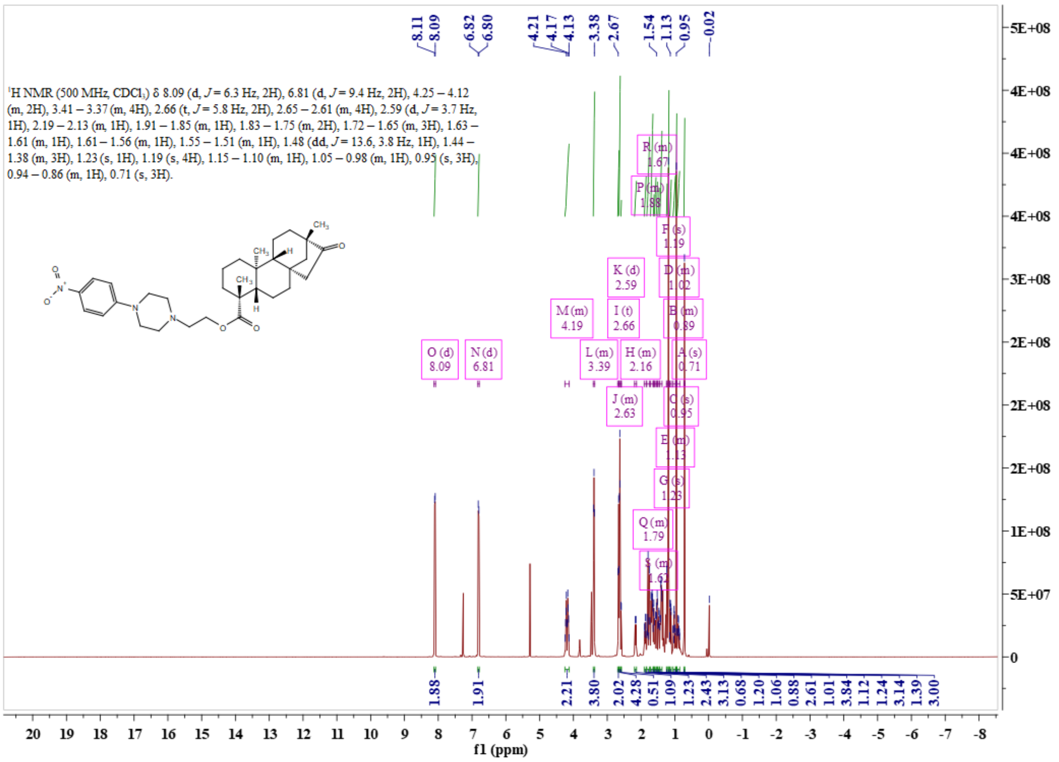

Figure S37. ^{1}H NMR spectrum of derivative **22.**

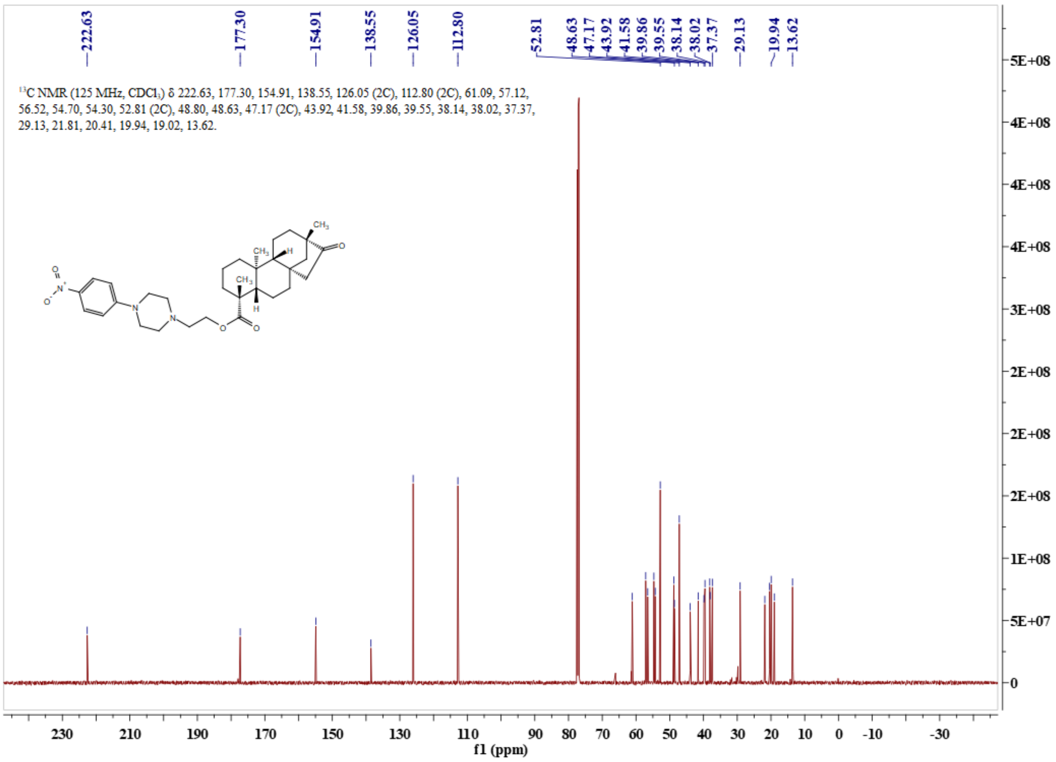

Figure S38. ^{13}C NMR spectrum of derivative **22.**
